# Factors predicting mortality in hospitalised HIV-negative children with lower-chest-wall indrawing pneumonia and implications for management

**DOI:** 10.1371/journal.pone.0297159

**Published:** 2024-03-11

**Authors:** Katherine E. Gallagher, Juliet O. Awori, Maria D. Knoll, Julia Rhodes, Melissa M. Higdon, Laura L. Hammitt, Christine Prosperi, Henry C. Baggett, W. Abdullah Brooks, Nicholas Fancourt, Daniel R. Feikin, Stephen R. C. Howie, Karen L. Kotloff, Milagritos D. Tapia, Orin S. Levine, Shabir A. Madhi, David R. Murdoch, Katherine L. O’Brien, Donald M. Thea, Vicky L. Baillie, Bernard E. Ebruke, Alice Kamau, David P. Moore, Lawrence Mwananyanda, Emmanuel O. Olutunde, Phil Seidenberg, Samba O. Sow, Somsak Thamthitiwat, J. Anthony G. Scott, for The PERCH Study Group

**Affiliations:** 1 Department of Infectious Disease Epidemiology, Faculty of Epidemiology and Population Health, London School of Hygiene and Tropical Medicine, London, United Kingdom; 2 Kenya Medical Research Institute-Wellcome Trust Research Programme, Kilifi, Kenya; 3 Department of International Health, International Vaccine Access Center, Johns Hopkins Bloomberg School of Public Health, Baltimore, Maryland, United States of America; 4 Global Disease Detection Center, Thailand Ministry of Public Health–US Centers for Disease Control and Prevention Collaboration, Nonthaburi, Thailand; 5 Division of Global Health Protection, Center for Global Health, Centers for Disease Control and Prevention, Atlanta, Georgia, United States of America; 6 Department of International Health, Johns Hopkins Bloomberg School of Public Health, Baltimore, Maryland, United States of America; 7 International Centre for Diarrhoeal Disease Research, Bangladesh (icddr,b), Dhaka and Matlab, Bangladesh; 8 Menzies School of Health Research, Charles Darwin University, Darwin, Australia; 9 Division of Viral Diseases, National Center for Immunizations and Respiratory Diseases, Centers for Disease Control and Prevention, Atlanta, Georgia, United States of America; 10 Medical Research Council Unit The Gambia at London School of Hygiene & Tropical Medicine, Basse, The Gambia; 11 Department of Paediatrics, University of Auckland, Auckland, New Zealand; 12 Department of Pediatrics, Center for Vaccine Development and Global Health, University of Maryland School of Medicine, Baltimore, Maryland, United States of America; 13 South African Medical Research Council: Vaccines and Infectious Diseases Analytics Research Unit, University of the Witwatersrand, Johannesburg, South Africa; 14 Department of Science and Technology/National Research Foundation: Vaccine Preventable Diseases Unit, University of the Witwatersrand, Johannesburg, South Africa; 15 Department of Pathology and Biomedical Sciences, University of Otago, Christchurch, New Zealand; 16 Microbiology Unit, Canterbury Health Laboratories, Christchurch, New Zealand; 17 Department of Global Health, Boston University School of Public Health, Boston, Massachusetts, United States of America; 18 Department of Paediatrics & Child Health, Chris Hani Baragwanath Academic Hospital and University of the Witwatersrand, Johannesburg, South Africa; 19 Right to Care-Zambia, Lusaka, Zambia; 20 Centre pour le Développement des Vaccins (CVD-Mali), Bamako, Mali; Projahnmo Research Foundation, BANGLADESH

## Abstract

**Introduction:**

In 2012, the World Health Organization revised treatment guidelines for childhood pneumonia with lower chest wall indrawing (LCWI) but no ‘danger signs’, to recommend home-based treatment. We analysed data from children hospitalized with LCWI pneumonia in the Pneumonia Etiology Research for Child Health (PERCH) study to identify sub-groups with high odds of mortality, who might continue to benefit from hospital management but may not be admitted by staff implementing the 2012 guidelines. We compare the proportion of deaths identified using the criteria in the 2012 guidelines, and the proportion of deaths identified using an alternative set of criteria from our model.

**Methods:**

PERCH enrolled a cohort of 2189 HIV-negative children aged 2–59 months who were admitted to hospital with LCWI pneumonia (without obvious cyanosis, inability to feed, vomiting, convulsions, lethargy or head nodding) between 2011–2014 in Kenya, Zambia, South Africa, Mali, The Gambia, Bangladesh, and Thailand. We analysed risk factors for mortality among these cases using predictive logistic regression. Malnutrition was defined as mid-upper-arm circumference <125mm or weight-for-age z-score <-2.

**Results:**

Among 2189 cases, 76 (3·6%) died. Mortality was associated with oxygen saturation <92% (aOR 3·33, 1·99–5·99), HIV negative but exposed status (4·59, 1·81–11·7), moderate or severe malnutrition (6·85, 3·22–14·6) and younger age (infants compared to children 12–59 months old, OR 2·03, 95%CI 1·05–3·93). At least one of three risk factors: hypoxaemia, HIV exposure, or malnutrition identified 807 children in this population, 40% of LCWI pneumonia cases and identified 86% of the children who died in hospital (65/76). Risk factors identified using the 2012 WHO treatment guidelines identified 66% of the children who died in hospital (n = 50/76).

**Conclusions:**

Although it focuses on treatment failure in hospital, this study supports the proposal for better risk stratification of children with LCWI pneumonia. Those who have hypoxaemia, any malnutrition or those who were born to HIV positive mothers, experience poorer outcomes than other children with LCWI pneumonia. Consistent identification of these risk factors should be prioritised and children with at least one of these risk factors should not be managed in the community.

## Introduction

Pneumonia is the cause of one in every eight deaths (13%) in children under five years of age globally. In sub-Saharan Africa and South-East Asia, 17% of child deaths are attributed to the syndrome. An estimated 760,000 (651,000–943,000) children between 1–59 months of age die annually due to pneumonia, making effective interventions to reduce pneumonia mortality of public health importance [1].

As a continuation of the WHO’s long-term strategy to combat acute respiratory illness, in 2005, the Integrated Management of Childhood Illness (IMCI) guidelines recommended community-based care with oral antibiotics for non-severe pneumonia cases and hospitalisation and parenteral antibiotics for any child presenting with difficulty breathing and lower chest wall-indrawing (LCWI), with or without danger signs (head nodding, central cyanosis, inability to feed/ drink, vomiting, convulsions, lethargy) [2, 3]. However, in many settings, successful referral for hospital-based care proved difficult. An observational study in Bangladesh [4] and a large trial of LCWI pneumonia in rural Pakistan [5] found significantly higher treatment failure in clusters where children presenting to outpatient clinics were referred to attend hospital some distance away (13%), compared to those given medication to take at home (8%). A review concluded community-based care could yield greater reductions in mortality than recommendations focused on case management in hospital [6]. In 2012, the guidelines for pneumonia cases presenting with LCWI but no danger signs (those danger signs listed above plus nasal flaring, grunting, cyanosis or hypoxaemia, very severe LCWI) were revised to recommend treatment with oral antibiotics at home rather than hospitalisation, in an effort to increase access to antibiotics and reduce any unnecessary risk of hospital-acquired infections [7, 8].

However, the studies used to support a change in recommendations included consistent utilisation of pulse oximetry on every child and active clinical follow-up of home-based care and do not necessarily represent routine community-based care in low-resource settings. Additionally, severe cases are more likely to attend hospital and controlling for case-mix on presentation to hospital in studies such as these, is challenging.

To implement the new recommendations, resources are needed to consistently access pulse oximetry, diagnose underlying conditions, recognise signs of severe respiratory distress, and follow-up treatment failures [9]. The 2012 guidelines identify the following indicators for admission: central cyanosis or hypoxemia, severe malnutrition, HIV exposure; other underlying conditions at triage may indicate the need for adapted treatment pathways e.g. prematurity (if still <6 months of age), congenital heart disease and abnormalities (e.g. Trisomy 21, glucose-6-phosphate dehydrogenase (G6PD) deficiency). Aside from severe malnutrition, many of these conditions are not routinely identified and/or require diagnostic resources that are often unavailable e.g. in Kenya, pulse oximetry measurements were only available in 49% of peadiatric admissions to 18 public hospitals between 2014 and 2021 [10]; only 9% of admissions to an emergency department were offered HIV testing [11]. Signs of severe respiratory distress, like severe LCWI, can be difficult to determine if the child is agitated in a busy primary health care setting [12].

Attendance of follow-up visits to assess treatment outcome relies on parental health seeking behaviour and treatment outcome relies heavily on adherence to oral antibiotics, which can be variable. In Malawi, 20% of children took fewer than the recommended 10 doses when being treated for fast-breathing pneumonia (without chest indrawing) and 9·5% took seven or fewer doses [13]. The recommendation for outpatient care of children with LCWI pneumonia has been controversial [14] and analyses of Kenyan children who were admitted to hospital under the 2005 guidelines, but may have been recommended for home care under the 2012 guidelines, found that 3% subsequently died [15].

We aimed to identify sub-groups of children with LCWI pneumonia, with high odds of mortality. We compare the proportion of deaths identified using the criteria for admission in the 2012 guidelines, and the proportion of deaths identified using an alternative set of criteria from our model. We studied this retrospectively among HIV-negative cases that were enrolled in the Pneumonia Etiology Research for Child Health (PERCH) study [16].

## Methods

### The study population

PERCH enrolled children aged 1–59 months who were admitted to hospital with severe or very severe pneumonia, according to the 2005 WHO guidelines [8], between 2011–2014, in seven countries (Kenya, Zambia, South Africa, Mali, The Gambia, Bangladesh, and Thailand) [17, 18]. ‘Severe’ cases had a history of/observed cough, or difficulty breathing, and LCWI; ‘very severe’ cases presented with cough or difficulty breathing and at least one danger sign: central cyanosis, inability to feed, vomiting everything, convulsions, lethargy or head nodding [18]. Cases were enrolled on any day of the week, 24 hours per day [16].

**Fig 1:**
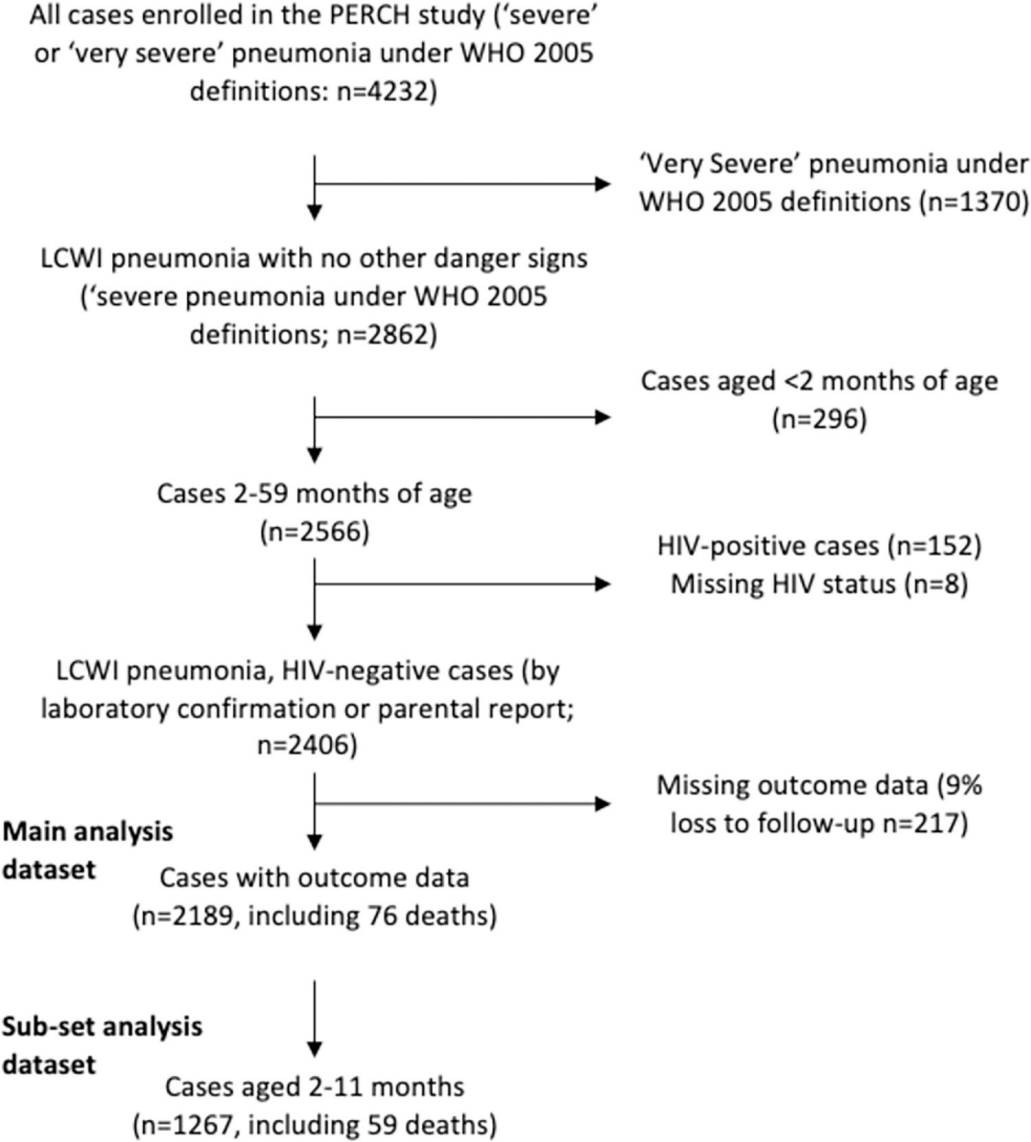
The study population.

As current World Health Organization (WHO) guidelines recommend hospitalisation of any child with suspected pneumonia who is confirmed HIV-positive or aged less than two months, these groups were excluded from the current analysis. We restricted this analysis to HIV-negative cases of ‘severe’ pneumonia i.e. ‘LCWI pneumonia’, without obvious cyanosis, reported inability to feed, vomiting, convulsions, lethargy or head nodding. We did not restrict the study population based on pulse oximetry measurements, HIV exposure or nutritional status in order to assess the predictive effect of these variables, which are inconsistently measured at the facility, on the odds of mortality. This was in order to assess risk factors for death among those who presented to hospital and were treated in hospital but who may have been treated at home under the WHO IMCI 2012 guidelines in the absence pulse oximetry, HIV exposure or nutritional assessment [7, 19]. Of the 2406 LCWI pneumonia cases in HIV-negative children between 2–59 months of age, 2189 (91%) had information on outcome collected at 30 days post-discharge (**Fig 1**). Although WHO guidelines recommend children with any other “underlying condition” (see the list in the introduction) are also admitted, independent of their pneumonia severity [7, 19], we included these children in the analysis, because this aspect of the guidelines, like pulse oximetry [10], is inconsistently implemented and the proportion of these cases who are correctly identified at admission in reality, is unclear.

### The study data

**Fig 2:**
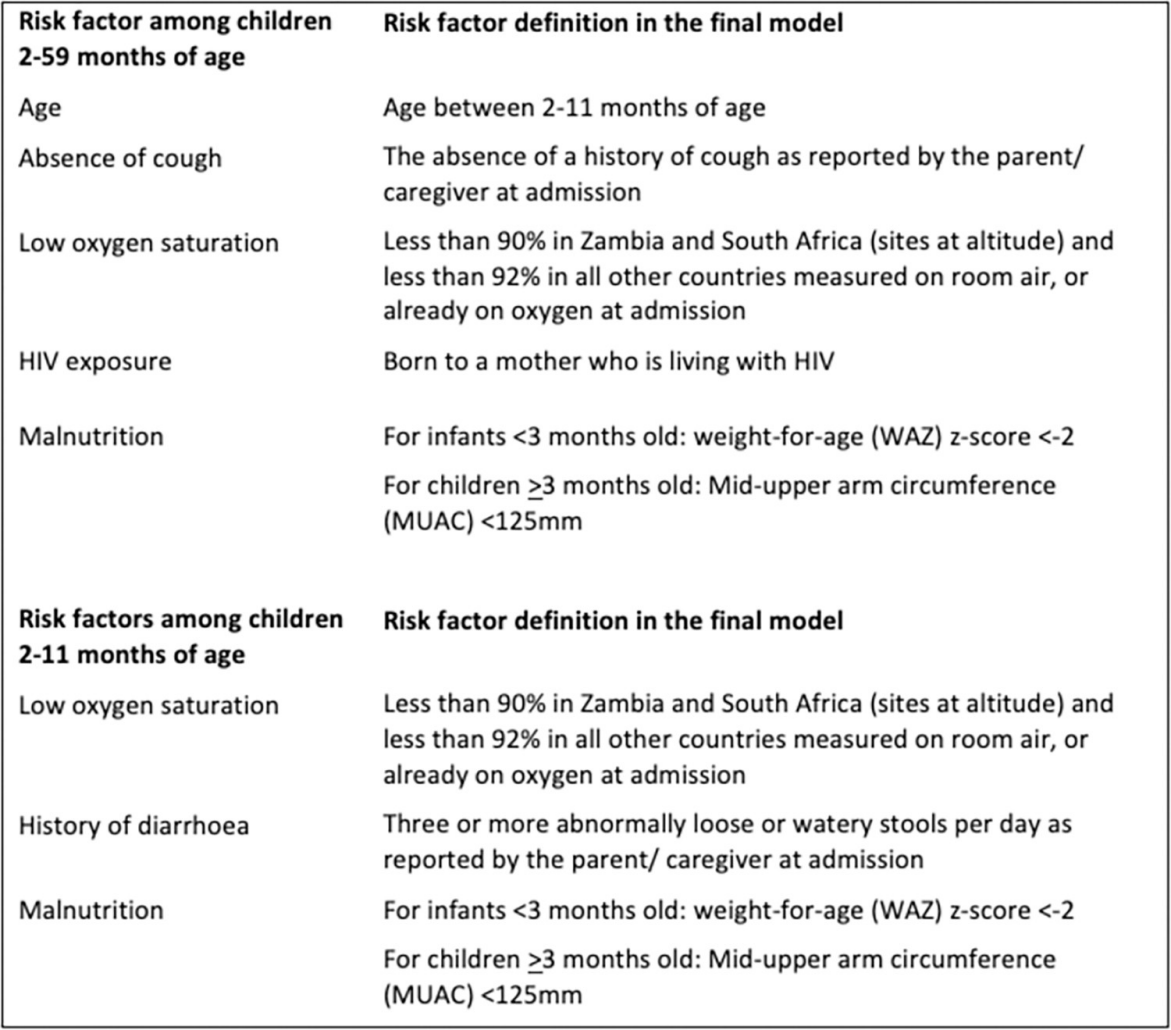
Definitions of the risk factors identified in the final models.

All sites collected sociodemographic information, medical history, birth history and vaccination status, anthropometry and signs on clinical examination (for key definitions see Fig 2). Standardized diagnostics and triage algorithms were used across all sites [17, 20]. Clinical data were obtained on admission to hospital, 24 and 48 hours post-admission, at discharge, and 30 days post-discharge. In this analysis ‘HIV-exposed’ indicates HIV-negative children born to mothers living with HIV.

The primary outcome of the analysis was ‘in-hospital death or death within seven days after discharge’, which was ascertained at a study visit 30 days post-discharge. Deaths within seven days after discharge were specific to the episode of pneumonia, in children who were discharged on parental request (n = 11).

### Predictive modelling

A total of 39 unique variables were assessed in univariable analyses (**S1 Table in S1 File**). Medication history, gestational age, and weak pulse were not assessed; more than 25% of these data were missing. A total of 189 (8.6%) children were put on oxygen at admission and did not have a measurement on room air; these children were classified pragmatically as hypoxaemic with 80–91% oxygen saturation. A sensitivity analysis excluding the children already on oxygen at admission was conducted.

Categorisations of continuous covariates were based on the cut-offs in WHO guidelines, clinical treatment algorithms, or a visual assessment of the continuous change in the odds of mortality across the values of the variable using cubic splines. Cubic splines modelled the non-linear change in odds of mortality [21] (**S1 Fig in S1 File**). All analyses were adjusted for country as a forced variable to control for clustering within the data. When there were multiple options in how to categorise specific variables, the p-value for the univariable association was used to choose the categorisation of a variable to include in the initial multivariable model. The other categorisations were then swapped into the final model to ensure the variable with the best fit had been chosen. If the p-values for variables or categorisations of variables were the same, the R-squared and the c-statistic of the model including each variable in turn informed the choice of variable to take forward to the multivariable regression model [22]. Interactions were assessed between all variables in the final multivariable model.

Variables potentially associated with mortality in univariable analyses (p-value <0.2) were included in a multivariable logistic regression model, controlling for country. Variables were iteratively removed from the model and only retained if they significantly improved model fit (likelihood-ratio test (LRT) p-value <0·05), using backwards stepwise regression [22]. In internal validation, the ability of the final full multivariable model to predict mortality was assessed using the area under the receiver operating curve (AUC or c-statistic) [23], bootstrapped with 200 repetitions to adjust for optimism [24]. As 38% of all fatal events occurred in the Zambian centre, we conducted a sensitivity analysis omitting the Zambian data.

We conducted a sub-group analysis restricted to infants aged 2–11 months (inclusive) using the same model building process. However, because of the smaller number of events in each country, random effects were used to control for clustering by country, rather than fitting fixed effects for each country. This meant that individual effect estimates for each country could not be calculated.

Finally, LCWI pneumonia cases were categorised into risk groups based on the presence or absence of combinations of the identified risk factors, in order to estimate 1) the proportion of children who would be admitted to hospital if management guidelines recommended it for their risk group, and 2) the proportion of children who subsequently died.

### Patient and public involvement

The analysis was conceptualised at a meeting of a WHO Working Group convened to discuss potential revisions to the guidelines for the management of pneumonia, in October 2018. The analysis directly addresses concerns raised by paediatricians about the current guidelines for the management of pneumonia [14, 15]. The results of this analysis have been disseminated to a group of stakeholders within the WHO Working Group and are intended to inform a revision of the guidelines. Patients themselves were not involved in the conceptualisation or implementation of this analysis.

### Ethical considerations

The study protocol, including the objectives of this analysis, was approved by the Institutional Review Boards at: Boston University (USA), the Faculte de Medecine, Pharmacie et Odontostomatologie (Mali), the Johns Hopkins Bloomberg School of Public Health (USA; SCC/EC1062, 3075), University of Maryland (USA); the US Centers for Disease Control and Prevention (Protocol 6076). The study was also approved by: the ERES Converge Ethical Review Committee (Zambia); The Gambian Government-Medical Research Council Joint Ethics Committee, The Kenya Medical Research Institute Scientific & Ethics review unit, Oxford University Tropical Research Ethics Committee; the Research Review Committee and the Ethical Review Committee of icddr,b (Bangladesh), the Thailand Ministry of Public Health Ethical Review Committee, The University of the Witwatersrand Human Research Ethics Committee (M101129). Written informed consent was obtained from parents/guardians prior to any study procedures.

## Results

### Risk factors for mortality in children aged 2–59 months of age

Among the 2189 HIV-negative cases of LCWI pneumonia in children aged 2–59 months, 76 (3·5%) died. The median length of hospital admission was 4 days (IQR 2–6; range 0–49). Almost half (47%) of those who died did so within 48 hours of admission (**Table 1**). Over 65% of deaths were in Zambia (38% of all deaths) and Mali (28% of all deaths), where a greater proportion of cases were infants, HIV-exposed, or had malnutrition or hypoxaemia than in other countries (**S2 Table in S1 File**). Pneumococcal (PCV) and Hib vaccine coverage was heterogeneous across the countries, only 0–2% of children were up to date with the PCV schedule for their age in Zambia, Thailand and Bangladesh. Thailand also had very low coverage of Hib vaccine (1.3%; **S2 Table in S1 File**).

**Table 1:** Time of death relative to hospital admission among HIV-negative cases of ‘lower-chest-wall indrawing’ (LCWI) pneumonia in the Pneumonia Etiology Research for Child Health study.

Time of death relative to admission	Died in hospital (N)	Died within 7 days after discharge ^1^(N)	Total	
			N	%
<2 days	35	1	36	47%
3–7 days	14	2	16	21%
8–14 days	11	4	15	20%
15–28 days	4	4	8	11%
>28 days	1	0	1	1%
Total	65	11	76	100%

^1^ Discharge diagnoses were pneumonia (8) septicaemia (1), complications of congenital heart disease (1) or missing (1). Median hospital stay was 13 days (range 2–21). Mean age was 7 months (range 2–23 months).

In univariable analyses, female sex, young age (2–11 months), low oxygen saturation, fever, reduced skin turgor, increased capillary refill time, a history of diarrhoea, more than 2 days of illness prior to presentation at hospital, HIV exposure, low anthropometric scores (weight-for-height, weight-for-age, height-for-age etc.) and mid-upper arm circumference (MUAC), low haemoglobin levels, and abnormal chest radiographs were associated (p<0·05) with increased odds of mortality, controlling for country. A medical history of cough was very common and associated with survival. The direction of the correlations between mortality and covariates in univariable analyses were similar across all countries (**S2 and S3 Tables in S1 File**).

Age (2–11 months), history of cough, low oxygen saturation, HIV exposure, and malnutrition were associated with mortality in the multivariable model, controlling for country (**Table 2**). Malnutrition was defined as moderate or severe i.e. MUAC <125mm in children aged ≥3 months and a weight-for-age z-score of <-2 among younger children (Fig 2). In HIV exposed children, the odds of death among those with very low weight-for-height z-scores were almost double those of HIV unexposed children (LRT p-interaction: 0·039), controlling for age, history of cough and oxygen saturation.

**Table 2:** Factors associated with mortality after presentation to hospital with ‘lower-chest-wall indrawing (non-severe) pneumonia’ in HIV-negative children aged 2–59 months enrolled in the Pneumonia Etiology Research for Child Health study.

Characteristic	Died							Unadjusted			Adjusted			
	No		Yes			Total		OR	95%CI	LRT p-value^a^	aOR	95%CI	LRT p-value^a^	
	n	%		n	%		n							
**All**	2113	**96·5**		76	**3·5**		2189							
**Country**														
South Africa	399	**98·3**		7	**1·7**		406	1			1			
Bangladesh	441	**99·6**		2	**0·5**		443	0·26	0·05–1·25		1·51	0·25–9·27		
Gambia	478	**99·0**		5	**1·0**		483	0·60	0·19–1·89		3·51	0·84–14·7		
Thailand	158	**97·5**		4	**2·5**		162	1·44	0·42–5·00		4·67	0·94–23·2		
Kenya	263	**97·1**		8	**3·0**		271	1·73	0·62–4·84	<0·0001	3·38	0·86–13·2	<0·0001	
Mali	251	**92·3**		21	**7·7**		272	4·77	2·00–11·4		9·23	2·78–30·7		
Zambia	123	**80·9**		29	**19·1**		152	13·4	5·75–31·4		24·2	8·42–69·4		
**Age**														
2–11 months	1208	**95·3**		59	**4·7**		1267	1·98	1·12–3·50	0·0143	2·03	1·05–3·93	0·0291	
12–59 months	905	**98·2**		17	**1·8**		922	1			1			
**Medical History**														
History of cough														
No	33	**80·5**		8	**19·5**		41	1		0·0060	1		0·045	
Yes	2080	**96·8**		68	**3·2**		2148	0·26	0·11–0·62		0·30	0·10–0·91		
HIV exposure^**b**^														
Unexposed	1716	**97·2**		50	**2·8**		1766	1		0·0096	1		0·101	
Exposed	183	**97·2**		18	**9·0**		201	2·63	1·27–5·43		1·92	0·88–4·19		
**Birth history/ Anthropometrics**													
MUAC/ Weight for age if <3months^**c**^													
Very low	128	**85·9**		21	**14·1**		149	7·07	3·69–13·5	<0·0001	4·51	2·16–9·38	<0·001
Low	176	**90·3**		19	**9·7**		195	4·47	2·38–8·40		3·90	1·97–7·71	
Normal-high	1760	**98·2**		33	**1·8**		1793	1			1		
**Signs on clinical examination**													
O2 saturation (80, 92)^**d**^														
<80%	72	**81·8**		16	**18·2**		88	7·15	3·47–14·7	<0·0001	6·51	2·82–15·0	0·0001	
80–91%	464	**94·7**		26	**5·3**		490	2·52	1·42–4·46		2·04	1·07–3·90		
> = 92% or > = 90% in SA/ ZAM	1574	**97·9**		34	**2·1**		1608	1			1			
**Interactions**														
HIV exposure & MUAC-WAZ^b^^,^^c^														
Unexposed, Normal/high MUAC-WAZ	1441	**98·8**		18	**1·2**		1459	1		<0.0001	1		<0·0001	
Unexposed, low MUAC-WAZ	144	**90·0**		16	**10·0**		160	7·14	3·41–15·0		6·85	3·22–14·6		
Unexposed, very low MUAC-WAZ	97	**86·6**		15	**13·4**		112	9·39	4·23–20·8		6·54	2·85–15·0		
Exposed, Normal/high MUAC-WAZ	140	**91·5**		13	**8·5**		153	4·83	1·98–11·8		4·59	1·81–11·7		
Exposed, low MUAC-WAZ	17	**89·5**		2	**10·5**		19	3·26	0·62–17·2		1·97	0·35–10·9		
Exposed, very low MUAC-WAZ	15	**88·2**		2	**11·8**		17	5·55	1·03–30·0		5·83	1·11–30·8		

Abbreviations and footnotes: aOR: adjusted Odds Ratio resulting from the multivariable logistic regression model; CI: Confidence Interval; LRT: Likelihood Ratio Test; MUAC: mid-upper arm circumference; O2: oxygen; OR: Odds Ratio; SA: South Africa; WAZ: weight-for-age z score; ZAM: Zambia.

The full list of factors analysed in univariable analyses are listed in S1 Table in S1 File.

^a^ p-values obtained from logistic regression likelihood ratio test, across all cases of LCWI pneumonia (Children presenting to hospital with cough or difficulty breathing (observed or history of) and observed LCWI but no danger signs using ‘country’ as a forced, indicator variable. During backwards regression, modelling covariates were removed from the model if they did not significantly improve the fit of the model to the data (p>0.05). When variables associated with the outcome in univariable analyses were added back into the model they did not significantly increase the fit of the model to the data. 1918 (88%) of 2189 observations were used in the final model. 1: reference category. The final adjusted model controlled for country, age, history of cough, HIV exposure and MUAC/ WAZ as an interaction, oxygen saturation.

^b^ HIV exposure is used to defined children who were HIV negative but born to mothers living with HIV. The interaction between HIV exposure and MUAC (or Weight for age if <3 months of age) was significant in the final model (p = 0.0044); however, inclusion of this interaction term presented the problem of sparse data with only 2 observations of death in the exposed low MUAC category, therefore estimates of the association between HIV exposure and MUAC-WAZ with the outcome in multivariable analyses are also included in the table to aid interpretation of the association.

^c^ WHO classifications: very low is <-3SDs from the mean, low is > = -3 SDs but <-2SDs from the mean; Normal to high is > = -2SDs from the mean.

^d^Oxygen saturation of 80–91% or children who were on oxygen at admission with no room air saturation measurement available A total of 437 (20%) of children had oxygen saturation <92% when measured on room air. A further 189 (8.6%) children were on oxygen at admission and did not have a measurement on room air; these children were classified as hypoxaemic with 80–91% oxygen saturation. Sensitivity analyses excluding those on oxygen at admission without oxygen saturation measurements on room air changed the effect estimates by less than 10%. Hypoxaemia was defined as oxygen saturation <90% in Zambia and South Africa (sites at altitude) and <92% in all other countries, or oxygen requirement on admission.

In sensitivity analyses, when data from children already on oxygen at admission were omitted, the same factors remained strongly associated with mortality in the multivariable model, with odds ratios that were higher, but within 10% of the reported values. When model building was repeated but restricted to data from countries other than Zambia; age, and HIV exposure were no longer significant factors in the multivariable analysis (**S4 Table in S1 File**). Hypoxaemia and malnutrition remained highly significant risk factors for death and a history of diarrhoea and nasal flaring on examination became significant risk factors. Due to the low number of events, there was limited power to detect other associations.

The final multivariable model across all countries predicted mortality well in internal validation (AUC adjusted for optimism: 0·81 (95%CI 0.77–0.87)). When applied to each country, the model performed poorly in The Gambia, Thailand, and Bangladesh (only 2–5 deaths in each setting; **S5 Table in S1 File**).

Approximately 16% of children presented with moderate or severe malnutrition; 12% of these children died, compared to 2% without malnutrition. A quarter of the children presented with oxygen saturation <92%, or were on oxygen on arrival and had no saturation measurement on ambient air; 7% of these hypoxaemic children died compared to 2% without hypoxaemia. The prevalence of HIV-exposure was 9% and 9% died, compared to 2·9% mortality among unexposed/unknown status children **(S6 Table in S1 File)**. The case fatality risk was increased in those with a combination of risk factors e.g. of 120 (6%) children with both hypoxaemia (<92%) and malnutrition, 24 (20%) died (**S6 Table in S1 File**).

**Fig 3:**
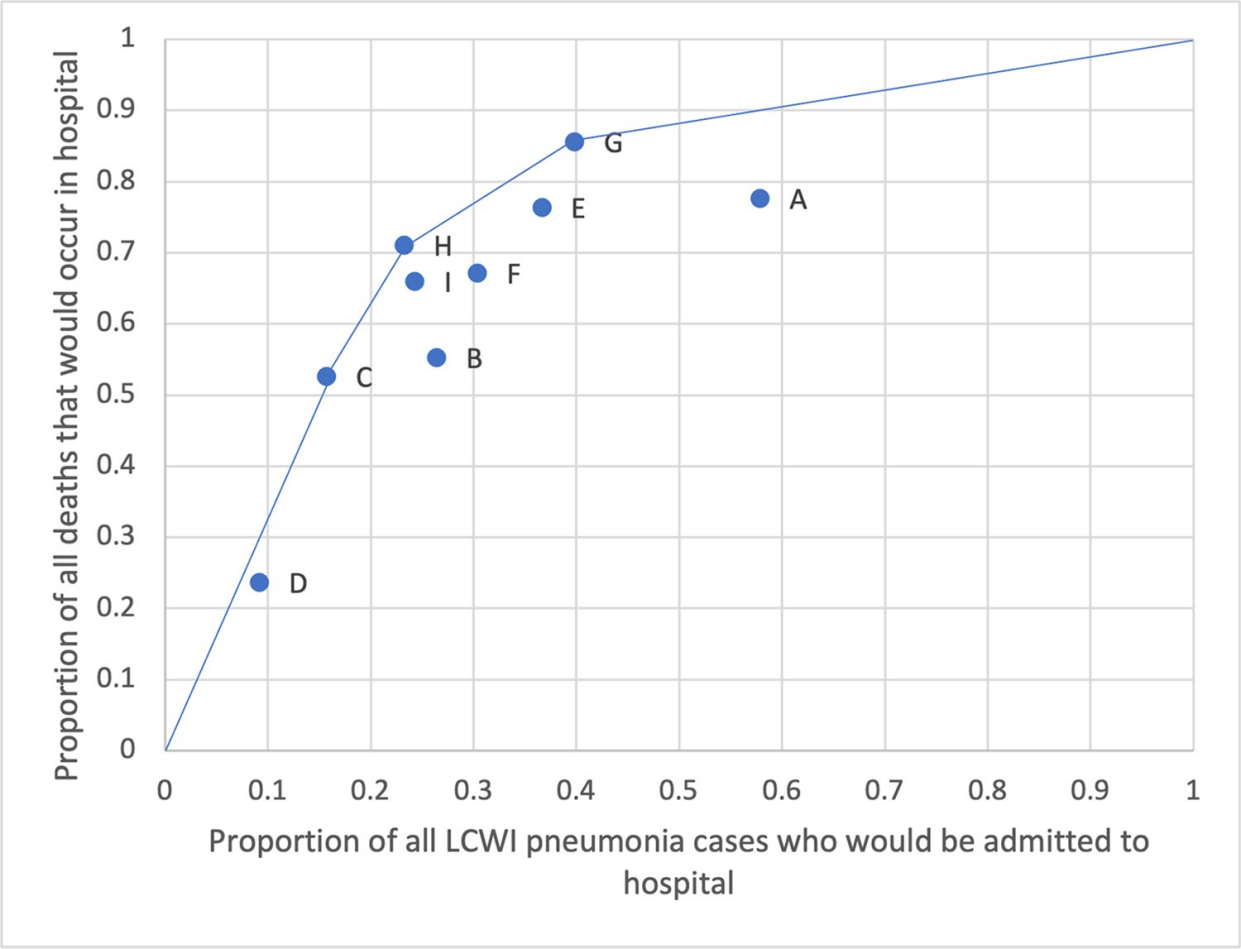
The proportion of HIV-negative LCWI pneumonia cases that would have been admitted to hospital and the proportion of all deaths that may have therefore occurred in hospital, under 8 different clinical management scenarios. The line links ‘efficient’ scenarios that minimise potentially unnecessary hospitalisation of cases. Scenario C: If all children with malnutrition (*low or very low mid-upper-arm circumference or weight-for-age scores)* were admitted to hospital but well-nourished children were treated at home: 16% of the PERCH LCWI cases would have been admitted, including 53% of the deaths. Scenario G: If LCWI pneumonia cases with *hypoxaemia*, *or HIV exposure*, *or low MUAC/WAZ* were admitted: 40% of the LCWI cases would have been admitted, including 86% of the deaths. Scenario H: If LCWI pneumonia cases with *low MUAC/WAZ or those HIV exposed* were admitted: 23% of all LCWI cases would have been admitted, including 71% of the deaths. Scenario I: If LCWI pneumonia cases with any *‘underlying condition’* as per IMCI guidelines were admitted i.e. those severely malnourished (WFH z-score <-3 regardless of age, or MUAC <115 mm for children ≥ 6 months, or pedal oedema on admission, or admission diagnosis of kwashiorkor), or children who were HIV exposed or premature and <6 months of age or with a diagnosis on admission of: heart disease, developmental delays, or congenital abnormalities (e.g. Trisomy 21, glucose-6-phosphate dehydrogenase (G6PD) deficiency): 24% of all LCWI cases would have been admitted, including 66% of the deaths. See S6 Table in S1 File for the complete list of scenarios examined.

Among 872 (40%) of children presenting with at least one of the following: malnutrition, hypoxeamia or HIV exposure, 65 (7·5%) died. Among 1317 children who presented with no malnutrition, hypoxeamia or HIV exposure, only 11 (0·8%) died (**Fig 3; S6 Table in S1 File**). Among 481 (24%) of children presenting with any underlying condition as defined by severe malnutrition (WFH z-score <-3 regardless of age, or MUAC <115 mm for children ≥ 6 months, or pedal edema on admission, or admission diagnosis of Kwashiorkor), or HIV exposure, or prematurity and less than 6 months of age, or a diagnosis at admission of: heart disease, including congenital heart conditions; developmental delays, including cerebral palsy; congenital abnormalities (e.g. Trisomy 21, G6PD), 50 (9.4%) died. Among the 1632 children who presented with no underlying conditions as defined by the above criteria, 26 (1.5%) of children died (**S6 and S7 Tables in S1 File**).

### Risk factors for mortality in infants 2–11 months of age

Of the 1267 infants, 702 (55%) were aged 2–5 months; mortality was 5·6% among infants aged 2–5 months and 3·5% among those aged 6–11 months. Sex, oxygen saturation, fever, skin turgor, prolonged capillary refill time, history of cough, history of diarrhoea, HIV exposure, low anthropometric scores, prematurity, low birthweight, and abnormal chest X-ray findings were associated with mortality in univariable analyses, with random effects controlling for clustering by country (**S8 Table in S1 File**). In multivariable analyses, oxygen saturation <92%, a history of diarrhoea, and malnutrition were significantly associated with mortality, controlling for clustering by country (**Table 3**).

**Table 3:** Factors associated with mortality after presentation to hospital with LCWI pneumonia in HIV-negative children aged 2–11 months.

Characteristic	Died					Unadjusted			Adjusted		
	No		Yes		Total	OR	95%CI	LRT p-value^a^	aOR	95%CI	LRT p-value^a^
	n	%	n	%	n						
All	1208	**95·3**	59	**4·7**	1267						
History of diarrhea											
No	1047	**96·4**	39	**3·6**	1086	1		0·0088	1		
Yes	161	**89·0**	20	**11·1**	181	2·31	1·26–4·23		2·40	1·24–4·64	0·0114
MUAC/ Weight for age if <3months^b^											
Very low	101	**87·1**	15	**12·9**	116	4·74	2·28–9·84	<0·0001	3·69	1·71–7·93	0·0003
Low	108	**88·5**	14	**11·5**	122	3·85	1·87–7·90		3·42	1·63–7·19	
Normal-high	958	**97·2**	28	**2·8**	986	1			1		
O2 saturation (80, 92)^c^											
<80%	48	**80·0**	12	**20·0**	60	7·31	3·12–17·1	<0·0001	6·84	2·78–16·8	0·0002
80–91%	291	**93·6**	20	**6·4**	311	2·41	1·24–4·66		2·14	1·07–4·29	
≥ 92% or ≥ 90% in SA/ ZAM	866	**97·0**	27	**3·0**	893	1			1		

Abbreviations and footnotes: aOR: adjusted Odds Ratio; CI: Confidence Interval; LRT: Likelihood Ratio Test; OR: Odds Ratio.

The full list of factors analysed in univariable analyses are listed in S1 Table in S1 File.

^a^ p-values obtained from logistic regression likelihood ratio test, across all cases of ‘severe’ pneumonia (WHO 2005 definitions: Children presenting to hospital with cough or difficulty breathing (observed or history of) and observed LCWI but no danger signs) using ‘country’ as a forced, indicator variable. During backwards regression modelling covariates were removed from the model if they did not significantly improve the fit of the model to the data (p>0.05). When variables associated with the outcome in univariable analyses were added back into the model they did not significantly increase the fit of the model to the data. A total of 1221 (96.4%) observations of 1267 were used in the final model. Due to the low number of events in each country, random effects were used to control for clustering by country, rather than calculating a fixed effect for each country, so individual ORs for each country were not calculable. 1: reference category.

^b^WHO classifications: very low is <-3SDs from the mean, low is > = -3 SDs but <-2SDs from the mean; Normal to high is > = -2SDs from the mean.

^c^ Oxygen saturation of 80–91% or on oxygen at admission with no oxygen saturation measurement on room air.

## Discussion

In this analysis of a large, prospectively collected dataset from seven countries, 3.6% of HIV-negative children aged 2–59 months died after admission to hospital with pneumonia defined by lower chest wall indrawing without obvious cyanosis, inability to feed, vomiting, convulsions, lethargy or head nodding (‘LCWI pneumonia’). We were able to assess a large number of potential predictors of mortality (**S9 Table in S1 File**). We identified five factors that predicted death: age (infancy), moderate or severe malnutrition (MUAC <125mm or low/very low WAZ or WFH), HIV exposure, hypoxaemia (oxygen saturation <92% or on supplementary oxygen at admission) and an absence of a history of cough. Many of these predictors have been identified in analyses of smaller datasets and many are already included in the 2012 WHO recommendations [15, 25]. The interaction between nutritional status and HIV exposure on treatment outcome is consistent with previous studies [26–29].

When considering how to refine recommendations for HIV-negative children presenting with LCWI pneumonia, admitting all children with at least one of three risk factors: hypoxaemia, HIV exposure, or any signs of malnutrition (807 children in this population, 40% of LCWI pneumonia cases) would result in 86% of the highest risk children being admitted (n = 65). Among all remaining children, the case fatality risk was 0·8% (**Fig 3**). Currently, children with MUAC <125mm or low anthropometric scores (i.e. not ‘very low’) are not included in the recommendations for hospitalisation, pulse oximetry to assess hypoxaemia and HIV exposure are included in the recommendations but are often not assessed [10].

The underlying factors indentified in the 2012 WHO guidelines that identify LCWI pneumonia cases in need of adapted treatment pathways i.e. severe malnutrition, HIV exposure, prematurity (if still <6 months of age), congenital heart disease and abnormalities (e.g. Trisomy 21, glucose-6-phosphate dehydrogenase (G6PD) deficiency), identified 24% of all children with LCWI pneumonia and captured 66% of all deaths. In reality, many of these underlying conditions are not identified in routine practice [30–33] and our model indicates that a simpler, feasible option may be to support focused, enhanced detection of any moderate-severe malnutrition, hypoxaemia and HIV exposure alone.

A quarter of children presented with hypoxaemia (oxygen saturation <92%) or were on oxygen at admission across the seven countries. When we excluded children who were placed on oxygen at admission, the prevalence of hypoxaemia was 19%, which is substantially higher than the prevalence (9%) in recent systematic reviews [34, 35]. The identification of oxygen saturation as a strong predictor of mortality, combined with evidence to suggest that oxygen therapy could improve outcomes, supports the expansion of pulse oximetry to triage patients for admission or referral [10, 34, 36–38].

The absence of cough was associated with mortality. Those presenting without a history of cough were predominantly infants (41% were between 2–5 months of age, 20% were 6–11 months of age) and had a higher prevalence of grunting (19% of those without cough displayed grunting compared to 14% of those with cough; p = 0·02). Grunting is a more severe sign of respiratory distress [39] that was not classified in PERCH as a danger sign on admission but is mentioned as a potential sign of severe respiratory distress in the 2012 WHO management guidelines. Cough may also be a marker of pneumonia aetiologies with milder prognoses e.g. RSV pneumonia, but we were unable to explore this hypothesis.

There are several limitations that merit discussion. The sample size available did not allow us to validate the models on a discrete dataset, and the capacity of these factors to predict poor outcome should therefore be validated on other datasets. In internal validation, HIV exposure, malnutrition and hypoxaemia predicted fatal outcome well (AUC adjusted for optimism: 0·81 for both models). However, the settings were highly heterogenous in the age of the cases, level of hypoxaemia, prevalence of wheeze, malnutrition and pneumococcal vaccine coverage and this hinders the generalisability of findings; any recommendations should take local context into account where possible. All of the study settings apart from Thailand had high vaccine coverage against *Heamophilus influenzae* type-b. There was little variation in *the direction* of the observed associations by country (**S3 Table in S1 File**), or the performance of the model by country (**S5 Table in S1 File**). However, we were unable to develop specific sets of risk factors for each country due to the low number of deaths. We assume children with the same characteristics as those who die are at high risk of death and may benefit from hospital care. As the factors were identified on presentation to hospital, and all the cases were admitted to hospital, we cannot infer how well these factors might predict poor outcome if applied to all community-based pneumonia cases, nor how these children may have fared with outpatient care at home. Additionally, we did not have data to control for the different level and quality of hospital-based care received by participants during their admission, across the different sites. This is likely to have influenced site-specific mortality risk, we controlled for ‘country’ to attempt to control for this but there is likely to be residual confounding.

A previous study in Kenya identified pallor, sex, dehydration, respiratory rate and temperature to be predictive of poor outcome [15]. Unfortunately information on pallor was not available in our dataset; the sex of the child was not significant in our final model [15] but, in univariable analyses, mortality was higher in girls than boys and hypoxaemia and very low weight-for-height scores on presentation to hospital were slightly more prevalent in girls than boys. Temperature, respiratory rate and capillary refill time [40] did not significantly improve the fit of our model in multivariable analyses. Approximately 80% of our study population had tachypnoea and fever, and therefore the data may have been too homogenous for these variables to statistically improve the fit of the multivariable model.

## Conclusions

Among 2189 HIV-negative children with LCWI pneumonia who were admitted to hospital in 7 heterogenous settings in Africa and Asia between 2011 and 2014, 76 (3.6%) died. Age (2–11 months), low oxygen saturation, malnutrition (MUAC <125mm or WAZ <-2), HIV exposure and the absence of cough were associated with death. These findings support the suggestion that a subset of children with LCWI pneumonia, who are hypoxaemic, malnourished, or have a history of HIV exposure, are at greater risk of dying and would benefit from admission to hospital [9]. Focused efforts to improve the consistent identification of these children at primary health care facilities for admission or referral to hospital may reduce mortality among those treated for LCWI pneumonia.

## Supporting information

S1 File (DOCX)

S2 File Inclusivity in global research. (DOCX)
