# Supplementary material for: Factors predicting mortality in hospitalised HIV-negative children with lower-chest-wall indrawing pneumonia and implications for management
Source: PLoS One. 2024 Mar 11;19(3):e0297159. doi: 10.1371/journal.pone.0297159 (PMC10927117; doi:10.1371/journal.pone.0297159)
Supplement: S1 File — (DOCX) [file pone.0297159.s001.docx]

**S1 Fig. Modelling the continuous, non-linear, variation in the odds of death across continuous values of covariates (a. Age, b. Oxygen saturation, c. respiratory rate, d. temperature at admission) using cubic splines, controlling for country**


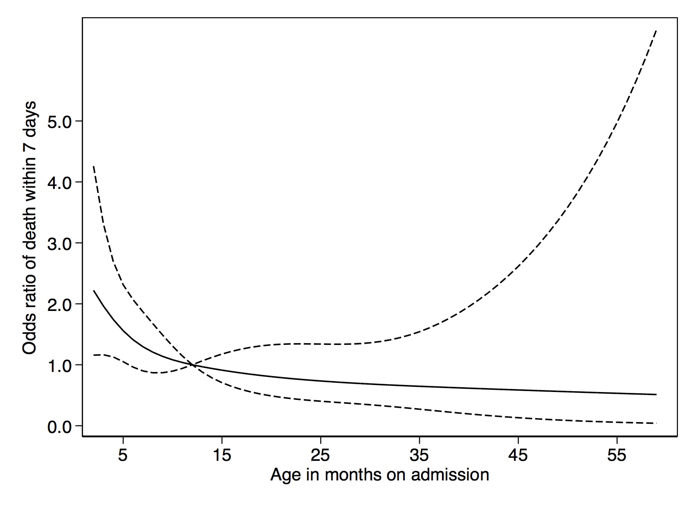

Footnotes: Solid line are smoothed odds ratios, dashed lines are 95%CI. A) Plot of the odds of death by age in months relative to 12 months of age; B) Plot of the odds of death by oxygen saturation, restricted to those children where oxygen saturation was measured on admission on room air (i.e. this does not include children whose oxygen saturation was measured while on oxygen), relative to the odds of death among those with 100% oxygen saturation; C) Plot of the odds of death by respiratory rate, relative to those with a respiratory rate of 40 bpm; D) Plot of the odds of death by temperature, relative to the odds of those with a temperature of 37.5 degrees

**S1 Table. Variables by stage of model development**

| **Covariates** | **39 variables in univariable analyses** | **23 variables assessed for inclusion in multivariable model** | **5 variables in final parsimonious multivariable model** | **3 variables in sub-analyses of infants only** |
| --- | --- | --- | --- | --- |
| Demographics | Age  Sex | Age  Sex | Age: 1-11mos; 12-59mos |  |
| Medical History | Difficulty breathing  Cough  Inability to feed  Ear discharge  Diarrhoea  Duration of illness  HIV exposure  DTP/PCV/Hib vaccination status  Premature/small at birth  Low birthweight | Cough  Inability to feed  Diarrhoea  Duration of illness  HIV exposure  Premature/small at birth  Low birthweight | History of Cough  HIV exposure | History of diarrhoea |
| Signs on clinical examination | O2 saturation  Respiratory rate/ tachypnoea  Tachycardia  Temperature/ fever  Cough  Wheeze  Grunting  Crackling  Nasal flaring  Deep breathing  Skin turgor  Capillary refill time  Weight for age  Height for age  Weight for height  Mid-upper Arm circumference (MUAC) for age  MUAC and/or WAZ  MUAC and/or WHZ  BMI for age | O2 saturation  Fever  Wheeze  Skin turgor  Capillary refill time  Weight for age  Height for age  Weight for height  Mid-upper Arm circumference (MUAC) for age  MUAC and/or WAZ  MUAC and/or WHZ  BMI for age | O2 saturation  MUAC and/or WAZ | O2 saturation  MUAC and/or WAZ |
| Diagnostics | Chest X-ray findings  Leucocytosis  Heamoglobin level  C-Reactive Protein level  Malaria | Chest X-ray findings  HB level |  |  |

**S2a Table. Distribution of risk factors by Country (2-59 months old)**

| **Characteristic** |  | |  | |  | |  | |  | |  | |  | |  |  |  |
| --- | --- | --- | --- | --- | --- | --- | --- | --- | --- | --- | --- | --- | --- | --- | --- | --- | --- |
|  | **Total** |  | | **Kenya** | | **Gambia** | | **Mali** | | **Zambia** | | **SA** | | **Thailand** | | **Bangladesh** |  |
|  | **n** | % | | n **(%)** | | n **(%)** | | n **(%)** | | n **(%^a^)** | | n **(%^a^)** | | n **(%^a^)** | | n **(%^a^)** | Chi2 p-value |
| **All** | 2189 |  | | 271 | | 483 | | 272 | | 152 | | 406 | | 162 | | 443 |  |
| **Age** |  |  | |  | |  | |  | |  | |  | |  | |  |  |
| 2-11 months | 1267 | 57.9 | | 154 (56.8) | | 299 (61.9) | | 170 (62.5) | | 116 (76.3) | | 277 (68.2) | | 53 (32.7) | | 198 (44.7) | <0.001 |
| 12-59 months | 922 | 42.1 | | 117 (43.2) | | 184 (38.1) | | 102 (37.5) | | 36 (23.7) | | 129 (31.8) | | 109 (67.3) | | 245 (55.3) |  |
| **Sex** |  |  | |  | |  | |  | |  | |  | |  | |  |  |
| Male | 1308 | 59.8 | | 160 (59.0) | | 308 (63.8) | | 148 (54.4) | | 78 (51.3) | | 218 (53.7) | | 106 (65.4) | | 290 (65.5) | <0.001 |
| Female | 881 | 40.3 | | 111 (41.0) | | 175 (36.2) | | 124 (45.6) | | 74 (48.7) | | 188 (46.3) | | 56 (34.6) | | 153 (34.5) |  |
| History of cough |  |  | |  | |  | |  | |  | |  | |  | |  |  |
| No | 41 | 1.9 | | 8 (3.0) | | 0 | | 18 (6.6) | | 6 (4.0) | | 6 (1.5) | | 3 (1.9) | | 0 | <0.001 |
| Yes | 2148 | 98.1 | | 263 (97.0) | | 483 (100) | | 254 (93.4) | | 146 (96.0) | | 400 (98.5) | | 159 (98.1) | | 443 (100) |  |
| HIV exposure |  |  | |  | |  | |  | |  | |  | |  | |  |  |
| Unexposed | 1766 | 89.8 | | 246 (100) | | 385 (99.5) | | 197 (98.0) | | 99 (67.4) | | 238 (62.0) | | 158 (99.4) | | 443 (100) | <0.001 |
| Exposed | 201 | 10.2 | | 0 | | 2 (0.5) | | 4 (2.0) | | 48 (32.7) | | 146 (38.0) | | 1 (0.6) | | 0 |  |
| MUAC/ Weight for age if <3months^7^ |  |  | |  | |  | |  | |  | |  | |  | |  |  |
| Very low | 149 | 7.0 | | 26 (9.7) | | 14 (2.9) | | 59 (21.8) | | 9 (6.2) | | 22 (6.0) | | 8 (5.0) | | 11 (2.5) | <0.001 |
| Low | 195 | 9.1 | | 42 (15.7) | | 29 (6.0) | | 40 (14.8) | | 21 (14.4) | | 30 (8.2) | | 7 (4.4) | | 26 (5.9) |  |
| Normal-high | 1793 | 83.9 | | 200 (75.6) | | 439 (91.1) | | 172 (63.5) | | 116 (79.5) | | 314 (85.8) | | 146 (90.7) | | 406 (91.7) |  |
| O2 saturation (80, 92) |  |  | |  | |  | |  | |  | |  | |  | |  |  |
| <80% | 88 | 4.0 | | 8 (3.0) | | 4 (0.8) | | 18 (6.6) | | 16 (10.6) | | 40 (9.9) | | 2 (1.2) | | 0 | <0.001 |
| 80-91%^2^ | 490 | 22.4 | | 58 (21.4) | | 24 (5.0) | | 84 (30.9) | | 30 (19.9) | | 245 (60.5) | | 31 (19.1) | | 18 (4.1) |  |
| >=92% or >=90% in SA/ ZAM | 1608 | 73.6 | | 205 (75.7) | | 454 (94.2) | | 170 (62.5) | | 105 (69.5) | | 120 (29.6) | | 129 (79.6) | | 425 (95.9) |  |
| PCV vaccination for age |  |  | |  | |  | |  | |  | |  | |  | |  |  |
| No vaccination | 934 | 43.8 | | 8 (3.0) | | 79 (17.1) | | 55 (20.5) | | 149 (98.0) | | 43 (11.3) | | 157 (99.4) | | 443 (100) | <0.001 |
| Partial vaccination | 248 | 11.6 | | 50 (18.6) | | 85 (18.4) | | 33 (12.3) | | 0 | | 80 (21.0) | | 0 | | 0 |  |
| Fully vaccinated/ up to date for age | 951 | 44.6 | | 211 (78.4) | | 298 (64.5) | | 180 (67.2) | | 3 (2.0) | | 258 (67.7) | | 1 (0.6) | | 0 |  |
| Hib vaccination for age |  |  | |  | |  | |  | |  | |  | |  | |  |  |
| No vaccination | 312 | 14.7 | | 3 (1.1) | | 69 (14.9) | | 21 (7.8) | | 3 (2.1) | | 44 (11.6) | | 155 (98.1) | | 17 (3.8) | <0.001 |
| Partial vaccination | 222 | 10.5 | | 19 (7.1) | | 72 (15.6) | | 29 (10.8) | | 9 (6.3) | | 75 (19.7) | | 1 (0.6) | | 17 (3.8) |  |
| Fully vaccinated/ up to date for age | 1589 | 74.9 | | 246 (91.8) | | 321 (69.5) | | 218 (81.3) | | 131 (91.6) | | 262 (68.8) | | 2 (1.3) | | 409 (92.3) |  |

^a^ Column percentages

**S2b Table. Distribution of mortality across risk factors, by country (2-59 months old)**

| **Characteristic** |  | | | | | | |  | | | | | |  | |  | | | | |  | | |  | | | |  | | | | |  |  |  |  |  |  |  |  |  |
| --- | --- | --- | --- | --- | --- | --- | --- | --- | --- | --- | --- | --- | --- | --- | --- | --- | --- | --- | --- | --- | --- | --- | --- | --- | --- | --- | --- | --- | --- | --- | --- | --- | --- | --- | --- | --- | --- | --- | --- | --- | --- |
|  |  |  |  | | | | **Overall mortality** | | | | **Kenya** | | | | | | **Gambia** | | | | | **Mali** | | | **Zambia** | | | | | **SA** | | | | | **Thailand** | | | | **Bangladesh** | | |
|  |  |  |  | | | | **n/N (%)** | | | | n/N **(%^a^)** | | | | | | n/N **(%^a^)** | | | | | n/N **(%^a^)** | | | n/N **(%^a^)** | | | | | n/N **(%^a^)** | | | | | n/N **(%^a^)** | | | | n/N **(%^a^)** | | |
| **All** |  |  |  | 76/ 2189 (3.5) | | | | | | | 8/271 (3.0) | | | | | | 5/483 (1.0) | | | | | 21/271 (7.7) | | | 29/152 (19.1) | | | | | 7/406 (1.7) | | | | | 4/162 (2.5) | | | | 2/443 (0.5) | | |
| **Age** |  |  |  |  | | | | | | |  | | | | | |  | | | | |  | | |  | | | | |  | | | | |  | | | |  | | |
| 2-11 months |  |  |  | 59/ 1267 (4.7) | | | | | | | 4/154 (2.6) | | | | | | 4/299 (1.3) | | | | | 16/170 (9.4) | | | 25/116 (21.6) | | | | | 6/277 (2.2) | | | | | 2/53 (3.8) | | | | 2/198 (1.0) | | |
| 12-59 months |  |  |  | 17/ 922 (1.8) | | | | | | | 4/117 (3.4) | | | | | | 1/184 (0.5) | | | | | 5/102 (4.9) | | | 4/36 (11.1) | | | | | 1/129 (0.8) | | | | | 2/109 (1.8) | | | | 0 | | |
| **Sex** |  |  |  |  | | | | | | |  | | | | | |  | | | | |  | | |  | | | | |  | | | | |  | | | |  | | |
| Male |  |  |  | 31/ 1308 (2.4) | | | | | | | 2/160 (1.3) | | | | | | 1/308 (0.3) | | | | | 10/148 (6.8) | | | 12/78 (15.4) | | | | | 3/218 (1.4) | | | | | 2/106 (1.9) | | | | 1/290 (0.3) | | |
| Female |  |  |  | 45/ 881 (5.1) | | | | | | | 6/111 (5.4) | | | | | | 4/175 (2.3) | | | | | 11/124 (8.9) | | | 17/74 (23.0) | | | | | 4/188 (2.1) | | | | | 2/56 (3.6) | | | | 1/153 (0.7) | | |
| **Medical History** |  |  |  |  | | | | | | |  | | | | | |  | | | | |  | | |  | | | | |  | | | | |  | | | |  | | |
| History of cough |  |  |  |  | | | | | | |  | | | | | |  | | | | |  | | |  | | | | |  | | | | |  | | | |  | | |
| No |  |  |  | 8/ 41 (19.5) | | | | | | | 0/8 (0) | | | | | |  | | | | | 2/18 (11.1) | | | 4/6 (66.7) | | | | | 0/6 | | | | | 2/3 (66.7) | | | |  | | |
| Yes |  |  |  | 68/ 2148 (3.2) | | | | | | | 8/263 (3.0) | | | | | | 5/483 (1.0) | | | | | 19/254 (7.5) | | | 25/146 (17.1) | | | | | 7/400 (1.8) | | | | | 2/159 (1.3) | | | | 2/443 (0.5) | | |
| HIV exposure |  |  |  |  | | | | | | |  | | | | | |  | | | | |  | | |  | | | | |  | | | | |  | | | |  | | |
| Unexposed |  |  |  | 50/ 1766 (2.8) | | | | | | | 6/246 (2.5) | | | | | | 5/385 (1.3) | | | | | 18/197 (9.1) | | | 13/99 (13.1) | | | | | 2/238 (0.8) | | | | | 4/158 (2.5) | | | | 2/443 (0.5) | | |
| Exposed |  |  |  | 18/ 201 (9.0) | | | | | | |  | | | | | | 0/2 (0) | | | | | 1/4 (25.0) | | | 13/48 (27.1) | | | | | 4/146 (2.7) | | | | | 0/1 (0) | | | |  | | |
| **Birth history/ Anthropometrics** | |  |  |  | | | | |  | | | |  | | | | | |  | | | |  | | | |  | | | | |  | | | | |  | | |  | |
| MUAC/ Weight for age if <3months^7^ |  |  |  | | |  | | | | | |  | | | | | |  | | | |  | | | |  | | | | |  | | | | |  | | |  | | |
| Very low |  |  |  | | | 21/ 149 (14.1) | | | | | | 2/26 (7.7) | | | | | | 0/14 (0) | | | | 15/59 (25.4) | | | | 4/9 (44.4) | | | | | 0/22 (0) | | | | | 0/8 (0) | | | 0/11 (0) | | |
| Low |  |  |  | | | 19/ 195 (9.7) | | | | | | 4/42 (9.5) | | | | | | 2/29 (6.9) | | | | 4/40 (10.0) | | | | 7/21 (33.3) | | | | | 1/30 (3.3) | | | | | 0/7 (0) | | | 1/26 (3.9) | | |
| Normal-high |  |  |  | | | 33/ 1793 (1.8) | | | | | | 2/200 (1.0) | | | | | | 3/439 (0.7) | | | | 2/172 (1.2) | | | | 17/116 (14.7) | | | | | 5/314 (1.6) | | | | | 3/146 (2.1) | | | 1/406 (0.3) | | |
| **Signs on clinical examination** | |  |  | |  | | | | |  | | | | |  | | | | |  | | | |  | | | | |  | | | | |  | | | |  | | |  |
| O2 saturation (80, 92) |  |  |  | | |  | | | | | |  | | | | | |  | | | |  | | | |  | | | | |  | | | | |  | | |  | | |
| <80% |  |  |  | | | 16/ 88 (18.2) | | | | | | 1/8 (12.5) | | | | | | 1/4 (25.0) | | | | 5/18 (27.8) | | | | 7/16 (43.8) | | | | | 2/40 (5.0) | | | | | 0/2 (0) | | |  | | |
| 80-91%^2^ |  |  |  | | | 26/ 490 (5.3) | | | | | | 3/58 (5.2) | | | | | | 1/24 (4.2) | | | | 8/84 (9.5) | | | | 8/30 (26.7) | | | | | 4/245 (1.6) | | | | | 2/31 (6.5) | | | 0/18 (0) | | |
| >=92% or >=90% in SA/ ZAM |  |  |  | | | 34/ 1608 (2.1) | | | | | | 4/205 (2.0) | | | | | | 3/454 (0.7) | | | | 8/170 (4.7) | | | | 14/105 (13.3) | | | | | 1/120 (0.8) | | | | | 2/129 (1.6) | | | 2/425 (0.5) | | |

^a^ Percentages represent the proportion in each category who died (the case fatality), ‘n’ is the number of deaths, ‘N’ is the total with the specified exposure.

**S3 Table. Factors associated with mortality after presentation to hospital with LCWI pneumonia in HIV-negative children aged 2-59 months**

| **Characteristic** | **Died** | | | | | | | | | | | **Unadjusted** | | | | | | **Adjusted** | | | | | |
| --- | --- | --- | --- | --- | --- | --- | --- | --- | --- | --- | --- | --- | --- | --- | --- | --- | --- | --- | --- | --- | --- | --- | --- |
|  | **No** | | | | | **Yes** | | | | **Total** | | **OR** | | **95%CI** | | **LRT**  **p-value^1^** | | **aOR** | | **95%CI** | | **LRT**  **p-value^1^** | |
|  | **n** | | | **%** | | **n** | | **%** | | **n** | |  | |  | |  |  |  | |  | |  |  |
| **All** | 2113 | | | **96.5** | | 76 | | **3.5** | | 2189 | |  | |  | |  | |  | |  | |  | |
| **Country** |  | | |  | |  | |  | |  | |  | |  | |  | |  | |  | |  | |
| Kenya | 263 | | | **97.1** | | 8 | | **3.0** | | 271 | | 1.73 | | 0.62-4.84 | | <0.0001 | | 3.38 | | 0.86-13.2 | | <0.0001 | |
| Gambia | 478 | | | **99.0** | | 5 | | **1.0** | | 483 | | 0.60 | | 0.19-1.89 | |  | | 3.51 | | 0.84-14.7 | |  | |
| Mali | 251 | | | **92.3** | | 21 | | **7.7** | | 272 | | 4.77 | | 2.00-11.4 | |  | | 9.23 | | 2.78-30.7 | |  | |
| Zambia | 123 | | | **80.9** | | 29 | | **19.1** | | 152 | | 13.4 | | 5.75-31.4 | |  | | 24.2 | | 8.42-69.4 | |  | |
| South Africa | 399 | | | **98.3** | | 7 | | **1.7** | | 406 | | 1 | |  | |  | | 1 | |  | |  | |
| Thailand | 158 | | | **97.5** | | 4 | | **2.5** | | 162 | | 1.44 | | 0.42-5.00 | |  | | 4.67 | | 0.94-23.2 | |  | |
| Bangladesh | 441 | | | **99.6** | | 2 | | **0.5** | | 443 | | 0.26 | | 0.05-1.25 | |  | | 1.51 | | 0.25-9.27 | |  | |
| **Age** |  | | |  | |  | |  | |  | |  | |  | |  | |  | |  | |  | |
| 2-11 months | 1208 | | | **95.3** | | 59 | | **4.7** | | 1267 | | 1.98 | | 1.12-3.50 | | 0.0143 | | 2.03 | | 1.05-3.93 | | 0.0291 | |
| 12-59 months | 905 | | | **98.2** | | 17 | | **1.8** | | 922 | | 1 | |  | |  | | 1 | |  | |  | |
| **Age (4)** |  | | |  | |  | |  | |  | |  | |  | |  | |  | |  | |  | |
| 2-5 months | 663 | | | **94.4** | | 39 | | **5.6** | | 702 | | 3.61 | | 1.25-10.5 | | 0.0318 | |  | |  | |  | |
| 6-11 months | 545 | | | **96.5** | | 20 | | **3.5** | | 565 | | 2.56 | | 0.85-7.74 | |  | |  | |  | |  | |
| 12-23 months | 564 | | | **97.8** | | 13 | | **2.3** | | 577 | | 1.94 | | 0.62-6.10 | |  | |  | |  | |  | |
| 24-59 months | 341 | | | **98.8** | | 4 | | **1.2** | | 345 | | 1 | |  | |  | |  | |  | |  | |
| **Sex** |  | | |  | |  | |  | |  | |  | |  | |  | |  | |  | |  | |
| Male | 1277 | | | **97.6** | | 31 | | **2.4** | | 1308 | | 1 | |  | | 0.0079 | |  | |  | |  | |
| Female | 836 | | | **94.9** | | 45 | | **5.1** | | 881 | | 1.91 | | 1.18-3.01 | |  | |  | |  | |  | |
| **Medical History** |  | | |  | |  | |  | |  | |  | |  | |  | |  | |  | |  | |
| History of difficulty breathing |  | | |  | |  | |  | |  | |  | |  | |  | |  | |  | |  | |
| No | 59 | | | **95.2** | | 3 | | **4.8** | | 62 | | 1 | |  | | 0.7443 | |  | |  | |  | |
| Yes | 2053 | | | **96.6** | | 73 | | **3.4** | | 2126 | | 0.81 | | 0.24-2.76 | |  | |  | |  | |  | |
| History of cough |  | | |  | |  | |  | |  | |  | |  | |  | |  | |  | |  | |
| No | 33 | | | **80.5** | | 8 | | **19.5** | | 41 | | 1 | |  | | 0.0060 | | 1 | |  | | 0.045 | |
| Yes | 2080 | | | **96.8** | | 68 | | **3.2** | | 2148 | | 0.26 | | 0.11-0.62 | |  | | 0.30 | | 0.10-0.91 | |  | |
| History of inability to feed |  | | |  | |  | |  | |  | |  | |  | |  | |  | |  | |  | |
| No | 2045 | | | **96.6** | | 71 | | **3.4** | | 2116 | | 1 | |  | | 0.1977 | |  | |  | |  | |
| Yes | 68 | | | **94.4** | | 4 | | **5.6** | | 72 | | 2.23 | | 0.72-6.93 | |  | |  | |  | |  | |
| History of ear discharge |  | | |  | |  | |  | |  | |  | |  | |  | |  | |  | |  | |
| No | 2102 | | | **96.6** | | 75 | | **3.5** | | 2177 | | 1 | |  | | 0.2651 | |  | |  | |  | |
| Yes | 10 | | | **90.9** | | 1 | | **9.1** | | 11 | | 4.26 | | 0.49-37.4 | |  | |  | |  | |  | |
| History of diarrhea |  | | |  | |  | |  | |  | |  | |  | |  | |  | |  | |  | |
| No | 1890 | | | **97.4** | | 50 | | **2.6** | | 1940 | | 1 | |  | | 0.0005 | |  | |  | |  | |
| Yes | 222 | | | **89.5** | | 26 | | **10.5** | | 248 | | 2.64 | | 1.56-4.47 | |  | |  | |  | |  | |
| Maximum duration of illness^6^ | | |  | |  | |  | |  | |  | |  | |  | |  | |  | |  | |  |
| 0-2 days | 779 | | | **98.2** | | 14 | | **1.8** | | 793 | | 1 | |  | |  | |  | |  | |  | |
| 3-5 days | 986 | | | **96.6** | | 35 | | **3.4** | | 1021 | | 1.97 | | 1.03-3.77 | | 0.0165 | |  | |  | |  | |
| >5 days | 341 | | | **92.9** | | 26 | | **7.1** | | 367 | | 2.66 | | 1.32-5.36 | |  | |  | |  | |  | |
| HIV exposure |  | | |  | |  | |  | |  | |  | |  | |  | |  | |  | |  | |
| Unexposed | 1716 | | | **97.2** | | 50 | | **2.8** | | 1766 | | 1 | |  | | 0.0096 | |  | |  | |  | |
| Exposed | 183 | | | **97.2** | | 18 | | **9.0** | | 201 | | 2.63 | | 1.27-5.43 | |  | |  | |  | |  | |
| **Vaccination status** |  | | |  | |  | |  | |  | |  | |  | |  | |  | |  | |  | |
| DTP vaccination status |  | | |  | |  | |  | |  | |  | |  | |  | |  | |  | |  | |
| None | 154 | | | **95.1** | | 8 | | **4.9** | | 162 | | 1 | |  | | 0.1203 | |  | |  | |  | |
| Partial | 270 | | | **96.8** | | 9 | | **3.2** | | 279 | | 0.39 | | 0.14-1.10 | |  | |  | |  | |  | |
| Full | 1626 | | | **96.7** | | 56 | | **3.3** | | 1682 | | 0.39 | | 0.17-0.90 | |  | |  | |  | |  | |
| Hib vaccination |  | | |  | |  | |  | |  | |  | |  | |  | |  | |  | |  | |
| None | 302 | | | **96.8** | | 10 | | **3.2** | | 312 | | 1 | |  | | 0.3843 | |  | |  | |  | |
| Partial | 217 | | | **97.8** | | 5 | | **2.3** | | 222 | | 0.43 | | 0.12-1.49 | |  | |  | |  | |  | |
| Full | 1531 | | | **96.4** | | 58 | | **3.7** | | 1589 | | 0.55 | | 0.22-1.38 | |  | |  | |  | |  | |
| PCV vaccination |  | | |  | |  | |  | |  | |  | |  | |  | |  | |  | |  | |
| None | 893 | | | **95.6** | | 41 | | **4.4** | | 934 | | 1 | |  | | 0.9193 | |  | |  | |  | |
| Partial | 242 | | | **97.6** | | 6 | | **2.4** | | 248 | | 0.87 | | 0.28-2.72 | |  | |  | |  | |  | |
| Full | 925 | | | **97.3** | | 26 | | **2.7** | | 951 | | 0.83 | | 0.35-1.98 | |  | |  | |  | |  | |
| **Birth history/ Anthropometrics** | |  | |  | |  | |  | |  | |  | |  | |  | |  | |  | |  | |
| Weight for height^7^ |  | | |  | |  | |  | |  | |  | |  | |  | |  | |  | |  | |
| Very low | 186 | | | **87.3** | | 27 | | **12.7** | | 213 | | 6.48 | | 3.56-11.8 | | <0.0001 | |  | |  | |  | |
| Low | 302 | | | **95.0** | | 16 | | **5.0** | | 318 | | 3.50 | | 1.80-6.78 | |  | |  | |  | |  | |
| Normal-high | 1585 | | | **98.3** | | 28 | | **1.7** | | 1613 | | 1 | |  | |  | |  | |  | |  | |
| Weight for age^7^ |  | | |  | |  | |  | |  | |  | |  | |  | |  | |  | |  | |
| Very low | 277 | | | **89.4** | | 33 | | **10.7** | | 310 | | 6.81 | | 3.79-12.2 | | <0.0001 | |  | |  | |  | |
| Low | 422 | | | **95.3** | | 21 | | **4.7** | | 443 | | 3.55 | | 1.88 -6.68 | |  | |  | |  | |  | |
| Normal-high | 1407 | | | **98.5** | | 22 | | **1.5** | | 1429 | | 1 | |  | |  | |  | |  | |  | |
| Height for age^7^ |  | | |  | |  | |  | |  | |  | |  | |  | |  | |  | |  | |
| Very low | 293 | | | **92.4** | | 24 | | **7.6** | | 317 | | 2.12 | | 1.17-3.85 | | 0.0485 | |  | |  | |  | |
| Low | 326 | | | **96.5** | | 12 | | **3.6** | | 338 | | 1.44 | | 0.72-2.87 | |  | |  | |  | |  | |
| Normal-high | 1464 | | | **97.6** | | 36 | | **2.4** | | 1500 | | 1 | |  | |  | |  | |  | |  | |
| MUAC for age^7^ |  | | |  | |  | |  | |  | |  | |  | |  | |  | |  | |  | |
| Very low | 93 | | | **83.8** | | 18 | | **16.2** | | 111 | | 7.22 | | 3.55-14.7 | | <0.0001 | |  | |  | |  | |
| Low | 147 | | | **91.9** | | 13 | | **8.1** | | 160 | | 3.31 | | 1.61-6.79 | |  | |  | |  | |  | |
| Normal-high | 1610 | | | **98.1** | | 31 | | **1.9** | | 1641 | | 1 | |  | |  | |  | |  | |  | |
| MUAC/ Weight for height if <3months^7^ |  | | |  | |  | |  | |  | |  | |  | |  | |  | |  | |  | |
| Very low | 112 | | | **84.9** | | 20 | | **15.2** | | 132 | | 6.46 | | 3.38-12.4 | | <0.0001 | |  | |  | |  | |
| Low | 169 | | | **91.9** | | 15 | | **8.2** | | 184 | | 3.15 | | 1.63-6.10 | |  | |  | |  | |  | |
| Normal-high | 1776 | | | **97.9** | | 38 | | **2.1** | | 1814 | | 1 | |  | |  | |  | |  | |  | |
| MUAC/ Weight for age if <3months^7^ |  | | |  | |  | |  | |  | |  | |  | |  | |  | |  | |  | |
| Very low | 128 | | | **85.9** | | 21 | | **14.1** | | 149 | | 7.07 | | 3.69-13.5 | | <0.0001 | |  | |  | |  | |
| Low | 176 | | | **90.3** | | 19 | | **9.7** | | 195 | | 4.47 | | 2.38-8.40 | |  | |  | |  | |  | |
| Normal-high | 1760 | | | **98.2** | | 33 | | **1.8** | | 1793 | | 1 | |  | |  | |  | |  | |  | |
| BMI for age^7^ |  | | |  | |  | |  | |  | |  | |  | |  | |  | |  | |  | |
| Very low | 210 | | | **87.9** | | 29 | | **12.1** | | 239 | | 6.11 | | 3.40-11.0 | | <0.0001 | |  | |  | |  | |
| Low | 288 | | | **95.4** | | 14 | | **4.6** | | 302 | | 3.10 | | 1.56-6.18 | |  | |  | |  | |  | |
| Normal-high | 1582 | | | **98.2** | | 29 | | **1.8** | | 1611 | | 1 | |  | |  | |  | |  | |  | |
| Premature or small at birth |  | | |  | |  | |  | |  | |  | |  | |  | |  | |  | |  | |
| No | 1605 | | | **97.3** | | 44 | | **2.7** | | 1649 | | 1 | |  | | 0.0051 | |  | |  | |  | |
| Yes | 497 | | | **94.7** | | 28 | | **5.3** | | 525 | | 2.12 | | 1.27-3.54 | |  | |  | |  | |  | |
| Birthweight |  | | |  | |  | |  | |  | |  | |  | |  | |  | |  | |  | |
| Normal-high (>2490g) | 1668 | | | **97.3** | | 47 | | **2.7** | | 1715 | | 1 | |  | | 0.0061 | |  | |  | |  | |
| Low (<= 2490g) | 426 | | | **94.5** | | 25 | | **5.5** | | 451 | | 2.14 | | 1.26-3.61 | |  | |  | |  | |  | |
| **Signs on clinical examination** | | |  | |  | |  | |  | |  | |  | |  | |  | |  | |  | |  |
| O2 saturation (60, 92) |  | | |  | |  | |  | |  | |  | |  | |  | |  | |  | |  | |
| <60% | 6 | | | **75.0** | | 2 | | **25.0** | | 8 | | 7.33 | | 1.09-49.1 | | <0.0001 | |  | |  | |  | |
| 60-91%^2^ | 530 | | | **93.0** | | 40 | | **7.0** | | 570 | | 3.25 | | 1.93-5.47 | |  | |  | |  | |  | |
| >= 92% (or 90% in SA/ZAM) | 1576 | | | **97.9** | | 34 | | **2.1** | | 1610 | | 1 | |  | |  | |  | |  | |  | |
| O2 saturation (80, 92) |  | | |  | |  | |  | |  | |  | |  | |  | |  | |  | |  | |
| <80% | 72 | | | **81.8** | | 16 | | **18.2** | | 88 | | 7.15 | | 3.47-14.7 | | <0.0001 | | 6.51 | | 2.82-15.0 | | 0.0001 | |
| 80-91%^2^ | 464 | | | **94.7** | | 26 | | **5.3** | | 490 | | 2.52 | | 1.42-4.46 | |  | | 2.04 | | 1.07-3.90 | |  | |
| >=92% or >=90% in SA/ ZAM | 1574 | | | **97.9** | | 34 | | **2.1** | | 1608 | | 1 | |  | |  | | 1 | |  | |  | |
| Hypoxaemia |  | | |  | |  | |  | |  | |  | |  | |  | |  | |  | |  | |
| No (>=90%) | 1651 | | | **97.8** | | 37 | | **2.2** | | 1688 | | 1 | |  | | <0.0001 | |  | |  | |  | |
| Yes (<90%)^3^ | 461 | | | **92.2** | | 39 | | **7.8** | | 500 | | 3.66 | | 2.18-6.16 | |  | |  | |  | |  | |
| Hypoxaemia |  | | |  | |  | |  | |  | |  | |  | |  | |  | |  | |  | |
| No | 1574 | | | **97.9** | | 34 | | **2.1** | | 1608 | | 1 | |  | | <0.0001 | |  | |  | |  | |
| Yes (<92%, or <90% in SA and ZAM)^4^ | 536 | | | **92.7** | | 42 | | **7.3** | | 578 | | 3.33 | | 1.99-5.99 | |  | |  | |  | |  | |
| Elevated respiratory rate for age^5^ |  | | |  | |  | |  | |  | |  | |  | |  | |  | |  | |  | |
| No | 257 | | | **96.3** | | 10 | | **3.8** | | 267 | | 1 | |  | | 0.5256 | |  | |  | |  | |
| Yes | 1849 | | | **96.6** | | 65 | | **3.4** | | 1914 | | 0.88 | | 0.44-1.80 | |  | |  | |  | |  | |
| Elevated respiratory rate (≥70 bpm) |  | | |  | |  | |  | |  | |  | |  | |  | |  | |  | |  | |
| No | 1741 | | | **97.1** | | 52 | | **2.9** | | 1793 | | 1 | |  | | 0.2938 | |  | |  | |  | |
| ≥70 bpm | 365 | | | **94.1** | | 23 | | **5.9** | | 388 | | 1.34 | | 0.78-2.31 | |  | |  | |  | |  | |
| Elevated heart rate for age^5^ |  | | |  | |  | |  | |  | |  | |  | |  | |  | |  | |  | |
| No | 1122 | | | **96.9** | | 36 | | **3.1** | | 1158 | | 1 | |  | | 0.7624 | |  | |  | |  | |
| Yes | 987 | | | **96.1** | | 40 | | **3.9** | | 1027 | | 0.93 | | 0.57-1.50 | |  | |  | |  | |  | |
| Fever (≥38°C or history of fever) |  | | |  | |  | |  | |  | |  | |  | |  | |  | |  | |  | |
| No | 360 | | | **96.3** | | 14 | | **3.7** | | 374 | | 1 | |  | | 0.5894 | |  | |  | |  | |
| Yes | 1753 | | | **96.6** | | 62 | | **3.4** | | 1815 | | 0.84 | | 0.45-1.58 | |  | |  | |  | |  | |
| Fever (≥39°C) |  | | |  | |  | |  | |  | |  | |  | |  | |  | |  | |  | |
| No | 1796 | | | **97.3** | | 50 | | **2.7** | | 1845 | | 1 | |  | | 0.0407 | |  | |  | |  | |
| Yes | 316 | | | **92.4** | | 26 | | **7.6** | | 342 | | 1.77 | | 1.04-3.01 | |  | |  | |  | |  | |
| Temperature |  | | |  | |  | |  | |  | |  | |  | |  | |  | |  | |  | |
| <35 | 1 | | | **100** | | 0 | | **0** | | 1 | | na | |  | |  | |  | |  | |  | |
| 35-38 | 1794 | | | **97.3** | | 50 | | **2.7** | | 1844 | |  | |  | |  | |  | |  | |  | |
| ≥39 | 316 | | | **92.4** | | 26 | | **7.6** | | 342 | |  | |  | |  | |  | |  | |  | |
| Cough (observed) |  | | |  | |  | |  | |  | |  | |  | |  | |  | |  | |  | |
| No | 513 | | | **95.2** | | 26 | | **4.8** | | 539 | | 1 | |  | | 0.3521 | |  | |  | |  | |
| Yes | 1595 | | | **97.0** | | 50 | | **3.0** | | 1645 | | 0.78 | | 0.46-1.31 | |  | |  | |  | |  | |
| Wheeze (on auscultation) |  | | |  | |  | |  | |  | |  | |  | |  | |  | |  | |  | |
| No | 1163 | | | **94.8** | | 64 | | **5.2** | | 1227 | | 1 | |  | | 0.1767 | |  | |  | |  | |
| Yes | 940 | | | **98.7** | | 12 | | **1.3** | | 952 | | 0.62 | | 0.31-1.27 | |  | |  | |  | |  | |
| Grunting (observed) |  | | |  | |  | |  | |  | |  | |  | |  | |  | |  | |  | |
| No | 1855 | | | **97.3** | | 52 | | **2.7** | | 1907 | | 1 | |  | | 0.3980 | |  | |  | |  | |
| Yes | 251 | | | **91.3** | | 24 | | **8.7** | | 275 | | 1.30 | | 0.71-2.37 | |  | |  | |  | |  | |
| Crackling/ crepitations (on auscultation) |  | | |  | |  | |  | |  | |  | |  | |  | |  | |  | |  | |
| No | 616 | | | **95.4** | | 30 | | **4.6** | | 646 | | 1 | |  | | 0.7423 | |  | |  | |  | |
| Yes | 1489 | | | **97.0** | | 46 | | **3.0** | | 1535 | | 1.09 | | 0.66-1.79 | |  | |  | |  | |  | |
| Nasal flaring |  | | |  | |  | |  | |  | |  | |  | |  | |  | |  | |  | |
| No | 980 | | | **97.8** | | 22 | | **2.2** | | 1002 | | 1 | |  | | 0.2581 | |  | |  | |  | |
| Yes | 1128 | | | **95.4** | | 54 | | **4.6** | | 1182 | | 1.37 | | 0.79-2.38 | |  | |  | |  | |  | |
| Deep Breathing |  | | |  | |  | |  | |  | |  | |  | |  | |  | |  | |  | |
| No | 1525 | | | **96.0** | | 63 | | **4.0** | | 1588 | | 1 | |  | | 0.3368 | |  | |  | |  | |
| Yes | 583 | | | **97.8** | | 13 | | **2.2** | | 596 | | 1.44 | | 0.69-3.00 | |  | |  | |  | |  | |
| Skin Turgor |  | | |  | |  | |  | |  | |  | |  | |  | |  | |  | |  | |
| Normal | 2058 | | | **96.9** | | 66 | | **3.1** | | 2124 | | 1 | |  | | 0.0018 | |  | |  | |  | |
| Reduced | 49 | | | **83.1** | | 10 | | **17.0** | | 59 | | 4.02 | | 1.81-8.93 | |  | |  | |  | |  | |
| Capillary refill time |  | | |  | |  | |  | |  | |  | |  | |  | |  | |  | |  | |
| <2 secs. | 1069 | | | **99.0** | | 11 | | **1.0** | | 1080 | | 1 | |  | | 0.0055 | |  | |  | |  | |
| 2-3 secs. | 816 | | | **95.0** | | 43 | | **5.0** | | 859 | | 2.44 | | 1.07-5.59 | |  | |  | |  | |  | |
| >3 secs. | 20 | | | **80.0** | | 5 | | **20.0** | | 25 | | 10.7 | | 2.72-41.7 | |  | |  | |  | |  | |
| Capillary refill time (2) |  | | |  | |  | |  | |  | |  | |  | |  | |  | |  | |  | |
| <2 secs | 1069 | | | **99.0** | | 11 | | **1.0** | | 1080 | | 1 | |  | | 0.0271 | |  | |  | |  | |
| ≥ 2 secs | 836 | | | **94.6** | | 48 | | **5.4** | | 884 | | 2.49 | | 1.09-5.70 | |  | |  | |  | |  | |
| **Diagnostics** |  | | |  | |  | |  | |  | |  | |  | |  | |  | |  | |  | |
| Chest Radiograph |  | | |  | |  | |  | |  | |  | |  | |  | |  | |  | |  | |
| Abnormal (consolidation/ infiltrate) | 1008 | | | **96.8** | | 33 | | **3.2** | | 1041 | | 1.93 | | 1.02-3.66 | |  | |  | |  | |  | |
| Normal | 895 | | | **98.5** | | 14 | | **1.5** | | 909 | | 1 | |  | | 0.0059 | |  | |  | |  | |
| Uninterpretable | 162 | | | **92.3** | | 13 | | **7.4** | | 175 | | 3.71 | | 1.65-8.33 | |  | |  | |  | |  | |
| Leukocytosis |  | | |  | |  | |  | |  | |  | |  | |  | |  | |  | |  | |
| No | 1042 | | | **96.0** | | 43 | | **4.0** | | 1085 | | 1 | |  | | 0.8416 | |  | |  | |  | |
| Yes | 907 | | | **96.8** | | 30 | | **3.3** | | 937 | | 1.01 | | 0.61-1.67 | |  | |  | |  | |  | |
| Anaemia/ Hb level^8^ |  | | |  | |  | |  | |  | |  | |  | |  | |  | |  | |  | |
| None (≥ 9.3 g/dl) | 1511 | | | **97.4** | | 41 | | **2.6** | | 1552 | | 1 | |  | | 0.0205 | |  | |  | |  | |
| Moderate (6-9.2) | 568 | | | **94.8** | | 31 | | **5.2** | | 599 | | 1.92 | | 1.15-3.21 | |  | |  | |  | |  | |
| Severe (<6 g/dl) | 34 | | | **89.5** | | 4 | | **10.5** | | 38 | | 2.95 | | 0.93-9.31 | |  | |  | |  | |  | |
| Anaemia (2) |  | | |  | |  | |  | |  | |  | |  | |  | |  | |  | |  | |
| None | 1511 | | | **97.4** | | 41 | | **2.6** | | 1552 | | 1 | |  | | 0.0069 | |  | |  | |  | |
| Moderate-severe | 602 | | | **94.5** | | 35 | | **5.5** | | 637 | | 2.00 | | 1.21-3.29 | |  | |  | |  | |  | |
| C-reactive protein |  | | |  | |  | |  | |  | |  | |  | |  | |  | |  | |  | |
| 0-40 mg/L  (~ 80% viral infections) | 1353 | | | **96.6** | | 48 | | **3.4** | | 1401 | | 1 | |  | | 0.6665 | |  | |  | |  | |
| >40 mg/L  (~ 80% bacterial infections) | 424 | | | **95.9** | | 18 | | **4.1** | | 442 | | 0.88 | | 0.50-1.57 | |  | |  | |  | |  | |
| Malaria test result^9^ |  | | |  | |  | |  | |  | |  | |  | |  | |  | |  | |  | |
| Negative | 16 | | | **100** | | 0 | | **0** | | 12 | | n/a | |  | |  | |  | |  | |  | |
| Positive | 2072 | | | **96.4** | | 76 | | **3.6** | | 2148 | |  | |  | |  | |  | |  | |  | |
| **Interactions** |  | | |  | |  | |  | |  | |  | |  | |  | |  | |  | |  | |
| HIV exposure & Weight for height (WFH) |  | | |  | |  | |  | |  | |  | |  | |  | |  | |  | |  | |
| Unexposed, Normal/high WFH | 1302 | | | **98.5** | | 20 | | **1.5** | | 1322 | | 1 | |  | | <0.0001 | |  | |  | |  | |
| Unexposed, low WFH | 239 | | | **96.4** | | 9 | | **3.6** | | 248 | | 2.51 | | 1.10-5.75 | |  | |  | |  | |  | |
| Unexposed, very low WFH | 148 | | | **90.2** | | 16 | | **9.8** | | 164 | | 5.15 | | 2.49-10.6 | |  | |  | |  | |  | |
| Exposed, Normal/high WFH | 136 | | | **95.8** | | 6 | | **4.3** | | 142 | | 1.89 | | 0.66-5.41 | |  | |  | |  | |  | |
| Exposed, low WFH | 16 | | | **72.7** | | 6 | | **27.3** | | 22 | | 29.3 | | 7.50-115 | |  | |  | |  | |  | |
| Exposed, very low WFH | 22 | | | **78.6** | | 6 | | **21.4** | | 28 | | 9.61 | | 2.99-30.9 | |  | |  | |  | |  | |
| HIV exposure & MUAC-WAZ^10^ |  | | |  | |  | |  | |  | |  | |  | |  | |  | |  | |  | |
| Unexposed, Normal/high MUAC-WAZ | 1441 | | | **98.8** | | 18 | | **1.2** | | 1459 | | 1 | |  | | <0.0001 | | 1 | |  | | <0.0001 | |
| Unexposed, low MUAC-WAZ | 144 | | | **90.0** | | 16 | | **10.0** | | 160 | | 7.14 | | 3.41-15.0 | |  | | 6.85 | | 3.22-14.6 | |  | |
| Unexposed, very low MUAC-WAZ | 97 | | | **86.6** | | 15 | | **13.4** | | 112 | | 9.39 | | 4.23-20.8 | |  | | 6.54 | | 2.85-15.0 | |  | |
| Exposed, Normal/high MUAC-WAZ | 140 | | | **91.5** | | 13 | | **8.5** | | 153 | | 4.83 | | 1.98-11.8 | |  | | 4.59 | | 1.81-11.7 | |  | |
| Exposed, low MUAC-WAZ | 17 | | | **89.5** | | 2 | | **10.5** | | 19 | | 3.26 | | 0.62-17.2 | |  | | 1.97 | | 0.35-10.9 | |  | |
| Exposed, very low MUAC-WAZ | 15 | | | **88.2** | | 2 | | **11.8** | | 17 | | 5.55 | | 1.03-30.0 | |  | | 5.83 | | 1.11-30.8 | |  | |
|  |  | | |  | |  | |  | |  | |  | |  | |  | |  | |  | |  | |

Abbreviations and footnotes: aOR: adjusted Odds Ratio; CI: Confidence Interval; LRT: Likelihood Ratio Test; OR: Odds Ratio; ref: reference category, WFH: weight-for-height.

^1^p-values obtained from logistic regression likelihood ratio test, across all cases of ‘severe’ pneumonia (WHO 2005 definitions: Children presenting to hospital with cough or difficulty breathing (observed or history of) and observed LCWI but no danger signs) using ‘country’ as a forced, indicator variable. During backwards regression modelling covariates were removed from the model if they did not significantly improve the fit of the model to the data (p>0.05). When variables associated with the outcome in univariable analyses were added back into the model they did not significantly increase the fit of the model to the data. 1918 of 2189 observations were used in the final model (88%).

^2^ Oxygen saturation of 80-91% or children who were on oxygen at admission with no room air saturation measurement available (189/2189 children (8.6%) were on oxygen at admission and therefore classified as hypoxaemic without room air oxygen saturation measurements)

^3^ Hypoxaemia was defined as <90% across all sites, or oxygen requirement on admission.

^4^ Hypoxaemia was defined as oxygen saturation <90% in Zambia and South Africa (sites at altitude) and <92% at all other sites, or oxygen requirement on admission.

^5^ Raised respiratory rate at admission defined as: < 2 months of age: ≥60 breaths/min, 2-11 months: ≥50 breaths/min, 12-59 months: ≥40 breaths/min. Elevated heart rate on admission was defined as: if 1-11 months of age: >160 bpm, 12-35 months: >150 bpm, 36-59 months: >140 bpm.

^6^ Maximum duration of illness with fever, cough, difficulty breathing or wheeze

^7^ WHO classifications: very low is <-3SDs away from the mean, low is >=-3 SDs but <-2SDs away from the mean; Normal to high is >=-2SDs.

^8^Anemia was classified as per the WHO guidelines for the management of common childhood illnesses (2013 ed.).

^9^Malaria tests were not conducted in South Africa, Bangladesh or Thailand where prevalence is thought to be <1%; missing results for these countries were imputed as negative for the purposes of these univariate analyses.

^10^ The interaction between HIV exposure and MUAC (or Weight for age if <3months of age) was significant in the final model (p=0.0044); however, presented the problem of sparse data with only 2 observations of death in the exposed low MUAC category, therefore the interaction term was excluded from the final multivariate model.

**S4 Table. A sensitivity analysis of factors associated with mortality after presentation to hospital with LCWI pneumonia in HIV-negative children aged 2-59 months, excluding the Zambian site**

| **Characteristic** | **Died** | | | | | | | | | | | **Unadjusted** | | | | | | **Adjusted** | | | | | |
| --- | --- | --- | --- | --- | --- | --- | --- | --- | --- | --- | --- | --- | --- | --- | --- | --- | --- | --- | --- | --- | --- | --- | --- |
|  | **No** | | | | | **Yes** | | | | **Total** | | **OR** | | **95%CI** | | **LRT**  **p-value^1^** | | **aOR** | | **95%CI** | | **LRT**  **p-value^1^** | |
|  | **n** | | | **%** | | **n** | | **%** | | **n** | |  | |  | |  |  |  | |  | |  |  |
| **All** | 1990 | | | **97.7** | | 47 | | **2.3** | | 2037 | |  | |  | |  | |  | |  | |  | |
| **Age** |  | | |  | |  | |  | |  | |  | |  | |  | |  | |  | |  | |
| 2-11 months | 1117 | | | **97.1** | | 34 | | **3.0** | | 1151 | | 1.91 | | 0.98-3.70 | | 0.0478 | |  | |  | |  | |
| 12-59 months | 873 | | | **98.5** | | 13 | | **1.5** | | 886 | | 1 | |  | |  | |  | |  | |  | |
| **Age (4)** |  | | |  | |  | |  | |  | |  | |  | |  | |  | |  | |  | |
| 2-5 months | 610 | | | **96.5** | | 22 | | **3.5** | | 632 | | 2.66 | | 0.89-7.98 | | 0.1839 | |  | |  | |  | |
| 6-11 months | 507 | | | **97.7** | | 12 | | **2.3** | | 519 | | 1.98 | | 0.63-6.29 | |  | |  | |  | |  | |
| 12-23 months | 544 | | | **98.4** | | 9 | | **1.6** | | 553 | | 1.38 | | 0.42-4.56 | |  | |  | |  | |  | |
| 24-59 months | 329 | | | **98.8** | | 4 | | **1.2** | | 333 | | 1 | |  | |  | |  | |  | |  | |
| **Sex** |  | | |  | |  | |  | |  | |  | |  | |  | |  | |  | |  | |
| Male | 1211 | | | **98.5** | | 19 | | **1,5** | | 1230 | | 1 | |  | | 0.0157 | |  | |  | |  | |
| Female | 779 | | | **96.5** | | 28 | | **3.5** | | 807 | | 2.07 | | 1.14-3.77 | |  | |  | |  | |  | |
| **Medical History** |  | | |  | |  | |  | |  | |  | |  | |  | |  | |  | |  | |
| History of difficulty breathing |  | | |  | |  | |  | |  | |  | |  | |  | |  | |  | |  | |
| No | 57 | | | **98.3** | | 1 | | **1.7** | | 58 | | 1 | |  | | 0.5251 | |  | |  | |  | |
| Yes | 1932 | | | **97.7** | | 46 | | **2.3** | | 1978 | | 0.81 | | 0.24-13.6 | |  | |  | |  | |  | |
| History of cough |  | | |  | |  | |  | |  | |  | |  | |  | |  | |  | |  | |
| No | 31 | | | **88.6** | | 4 | | **11.4** | | 35 | | 1 | |  | | 0.1260 | |  | |  | |  | |
| Yes | 1959 | | | **97.9** | | 43 | | **2.2** | | 2002 | | 0.38 | | 0.12-1.17 | |  | |  | |  | |  | |
| History of inability to feed |  | | |  | |  | |  | |  | |  | |  | |  | |  | |  | |  | |
| No | 1924 | | | **97.9** | | 42 | | **2.1** | | 1966 | | 1 | |  | | 0.1020 | |  | |  | |  | |
| Yes | 66 | | | **94.3** | | 4 | | **5.7** | | 70 | | 2.87 | | 0.91-9.05 | |  | |  | |  | |  | |
| History of ear discharge |  | | |  | |  | |  | |  | |  | |  | |  | |  | |  | |  | |
| No | 1979 | | | **97.7** | | 46 | | **2.3** | | 2025 | | 1 | |  | | 0.2651 | |  | |  | |  | |
| Yes | 10 | | | **90.9** | | 1 | | **9.1** | | 11 | | 4.26 | | 0.49-37.4 | |  | |  | |  | |  | |
| History of diarrhea |  | | |  | |  | |  | |  | |  | |  | |  | |  | |  | |  | |
| No | 1788 | | | **98.2** | | 32 | | **1.8** | | 1820 | | 1 | |  | | 0.0105 | | 1 | |  | | 0.0555 | |
| Yes | 201 | | | **93.1** | | 15 | | **6.9** | | 216 | | 2.47 | | 1.28-4.79 | |  | | 2.06 | | 1.01-4.23 | |  | |
| Maximum duration of illness^6^ | | |  | |  | |  | |  | |  | |  | |  | |  | |  | |  | |  |
| 0-2 days | 735 | | | **99.2** | | 6 | | **0.8** | | 741 | | 1 | |  | |  | |  | |  | |  | |
| 3-5 days | 939 | | | **97.2** | | 27 | | **2.8** | | 966 | | 3.06 | | 1.24-7.55 | | 0.0157 | |  | |  | |  | |
| >5 days | 309 | | | **95.7** | | 14 | | **4.3** | | 323 | | 3.43 | | 1.25-9.39 | |  | |  | |  | |  | |
| HIV exposure |  | | |  | |  | |  | |  | |  | |  | |  | |  | |  | |  | |
| Unexposed | 1630 | | | **97.8** | | 37 | | **2.2** | | 1667 | | 1 | |  | | 0.1026 | |  | |  | |  | |
| Exposed | 148 | | | **96.7** | | 5 | | **3.3** | | 153 | | 3.09 | | 0.82-11.6 | |  | |  | |  | |  | |
| **Vaccination status** |  | | |  | |  | |  | |  | |  | |  | |  | |  | |  | |  | |
| DTP vaccination status |  | | |  | |  | |  | |  | |  | |  | |  | |  | |  | |  | |
| None | 152 | | | **95.6** | | 7 | | **4** | | 159 | | 2.71 | | 1.12-6.59 | | 0.1263 | |  | |  | |  | |
| Partial | 254 | | | **97.7** | | 6 | | **2.3** | | 260 | | 1.20 | | 0.48-2.96 | |  | |  | |  | |  | |
| Full | 1530 | | | **98.0** | | 31 | | **2.0** | | 1561 | | 1 | |  | |  | |  | |  | |  | |
| Hib vaccination |  | | |  | |  | |  | |  | |  | |  | |  | |  | |  | |  | |
| None | 300 | | | **97.1** | | 9 | | **2.9** | | 309 | | 1.92 | | 0.71-5.21 | | 0.4760 | |  | |  | |  | |
| Partial | 208 | | | **97.7** | | 5 | | **2.4** | | 213 | | 1.20 | | 0.45-3.20 | |  | |  | |  | |  | |
| Full | 1428 | | | **97.9** | | 30 | | **2.1** | | 1458 | | 1 | |  | |  | |  | |  | |  | |
| PCV vaccination |  | | |  | |  | |  | |  | |  | |  | |  | |  | |  | |  | |
| None | 773 | | | **98.5** | | 12 | | **1.5** | | 785 | | 1.08 | | 0.43-2.73 | | 0.9872 | |  | |  | |  | |
| Partial | 242 | | | **97.6** | | 6 | | **2.4** | | 248 | | 1.02 | | 0.41-2.55 | |  | |  | |  | |  | |
| Full | 922 | | | **97.3** | | 26 | | **2.7** | | 948 | | 1 | |  | |  | |  | |  | |  | |
| **Birth history/ Anthropometrics** | |  | |  | |  | |  | |  | |  | |  | |  | |  | |  | |  | |
| Weight for height^7^ |  | | |  | |  | |  | |  | |  | |  | |  | |  | |  | |  | |
| Very low | 172 | | | **89.1** | | 21 | | **10.9** | | 193 | | 9.28 | | 4.45-19.3 | | <0.0001 | |  | |  | |  | |
| Low | 293 | | | **96.7** | | 10 | | **3.3** | | 303 | | 3.57 | | 1.54-8.30 | |  | |  | |  | |  | |
| Normal-high | 1489 | | | **99.1** | | 13 | | **0.9** | | 1502 | | 1 | |  | |  | |  | |  | |  | |
| Weight for age^7^ |  | | |  | |  | |  | |  | |  | |  | |  | |  | |  | |  | |
| Very low | 262 | | | **92.3** | | 22 | | **7.8** | | 284 | | 7.27 | | 3.51-15.1 | | <0.0001 | |  | |  | |  | |
| Low | 399 | | | **96.8** | | 13 | | **3.2** | | 412 | | 3.99 | | 1.79-8.90 | |  | |  | |  | |  | |
| Normal-high | 1323 | | | **99.1** | | 12 | | **0.9** | | 1335 | | 1 | |  | |  | |  | |  | |  | |
| Height for age^7^ |  | | |  | |  | |  | |  | |  | |  | |  | |  | |  | |  | |
| Very low | 250 | | | **96.9** | | 8 | | **3.10** | | 258 | | 1.83 | | 0.80-4.20 | | 0.2489 | |  | |  | |  | |
| Low | 307 | | | **97.2** | | 9 | | **2.9** | | 316 | | 1.65 | | 0.75-3.61 | |  | |  | |  | |  | |
| Normal-high | 1407 | | | **98.1** | | 27 | | **1.9** | | 1434 | | 1 | |  | |  | |  | |  | |  | |
| MUAC/ Weight for age if <3months^7^ |  | | |  | |  | |  | |  | |  | |  | |  | |  | |  | |  | |
| Very low | 128 | | | **87.9** | | 17 | | **12.1** | | 140 | | 8.51 | | 3.99-18.1 | | <0.0001 | | 7.87 | | 3.61-17.1 | | <0.0001 | |
| Low | 162 | | | **93.1** | | 12 | | **6.9** | | 174 | | 5.86 | | 2.65-12.9 | |  | | 5.24 | | 2.34-11.7 | |  | |
| Normal-high | 1661 | | | **99.1** | | 16 | | **1.0** | | 1677 | | 1 | |  | |  | | 1 | |  | |  | |
| BMI for age^7^ |  | | |  | |  | |  | |  | |  | |  | |  | |  | |  | |  | |
| Very low | 196 | | | **89.9** | | 22 | | **10.1** | | 218 | | 8.42 | | 4.07-17.4 | | <0.0001 | |  | |  | |  | |
| Low | 279 | | | **96.9** | | 9 | | **3.1** | | 288 | | 3.34 | | 1.40-7.95 | |  | |  | |  | |  | |
| Normal-high | 1486 | | | **99.1** | | 13 | | **0.9** | | 1499 | | 1 | |  | |  | |  | |  | |  | |
| Premature or small at birth |  | | |  | |  | |  | |  | |  | |  | |  | |  | |  | |  | |
| No | 1509 | | | **98.2** | | 27 | | **1.8** | | 1536 | | 1 | |  | | 0.0526 | |  | |  | |  | |
| Yes | 470 | | | **96.7** | | 16 | | **3.3** | | 486 | | 1.93 | | 1.01-3.67 | |  | |  | |  | |  | |
| Birthweight |  | | |  | |  | |  | |  | |  | |  | |  | |  | |  | |  | |
| Normal-high (>2490g) | 1568 | | | **98.2** | | 29 | | **1.82** | | 1597 | | 1 | |  | | 0.0682 | |  | |  | |  | |
| Low (<= 2490g) | 403 | | | **96.6** | | 14 | | **3.4** | | 417 | | 1.89 | | 0.98-3.66 | |  | |  | |  | |  | |
| **Signs on clinical examination** | | |  | |  | |  | |  | |  | |  | |  | |  | |  | |  | |  |
| O2 saturation (60, 92) |  | | |  | |  | |  | |  | |  | |  | |  | |  | |  | |  | |
| <60% | 5 | | | **100** | | 0 | | **0** | | 5 | | - | | - | | <0.0002 | |  | |  | |  | |
| 60-91%^2^ | 500 | | | **94.9** | | 27 | | **5.1** | | 527 | | 3.48 | | 1.80-6.72 | |  | |  | |  | |  | |
| >= 92% (or 90% in SA/ZAM) | 1485 | | | **98.7** | | 20 | | **1.3** | | 1505 | | 1 | |  | |  | |  | |  | |  | |
| O2 saturation (80, 92) |  | | |  | |  | |  | |  | |  | |  | |  | |  | |  | |  | |
| <80% | 63 | | | **87.5** | | 9 | | **12.5** | | 72 | | 8.98 | | 3.60-22.4 | | <0.0001 | |  | |  | |  | |
| 80-91%^2^ | 442 | | | **96.1** | | 18 | | **3.9** | | 460 | | 2.68 | | 1.32-5.46 | |  | |  | |  | |  | |
| >=92% or >=90% in SA/ ZAM | 1483 | | | **98.7** | | 20 | | **1.3** | | 1503 | | 1 | |  | |  | |  | |  | |  | |
| Hypoxaemia |  | | |  | |  | |  | |  | |  | |  | |  | |  | |  | |  | |
| No (>=90%) | 1560 | | | **98.6** | | 23 | | **1.5** | | 1583 | | 1 | |  | | 0.0001 | | 1 | |  | | 0.0007 | |
| Yes (<90%)^3^ | 430 | | | **94.7** | | 24 | | **5.3** | | 454 | | 4.03 | | 2.07-7.83 | |  | | 3.36 | | 1.68-6.74 | |  | |
| Hypoxaemia |  | | |  | |  | |  | |  | |  | |  | |  | |  | |  | |  | |
| No | 1483 | | | **98.7** | | 20 | | **1.3** | | 1503 | | 1 | |  | | <0.0002 | |  | |  | |  | |
| Yes (<92%, or <90% in SA and ZAM)^4^ | 505 | | | **94.9** | | 27 | | **5.1** | | 532 | | 3.46 | | 1.79-6.67 | |  | |  | |  | |  | |
| Elevated respiratory rate for age^5^ |  | | |  | |  | |  | |  | |  | |  | |  | |  | |  | |  | |
| No | 245 | | | **97.2** | | 7 | | **2.8** | | 252 | | 1 | |  | | 0.7224 | |  | |  | |  | |
| Yes | 1740 | | | **97.8** | | 39 | | **2.2** | | 1779 | | 0.86 | | 0.37-1.98 | |  | |  | |  | |  | |
| Elevated respiratory rate (≥70 bpm) |  | | |  | |  | |  | |  | |  | |  | |  | |  | |  | |  | |
| No | 1662 | | | **97.7** | | 40 | | **2.4** | | 1702 | | 1 | |  | | 0.4232 | |  | |  | |  | |
| ≥70 bpm | 323 | | | **98.2** | | 6 | | **1.8** | | 329 | | 0.71 | | 0.29-1.70 | |  | |  | |  | |  | |
| Elevated heart rate for age^5^ |  | | |  | |  | |  | |  | |  | |  | |  | |  | |  | |  | |
| No | 1076 | | | **97.9** | | 23 | | **2.1** | | 1099 | | 1 | |  | | 0.9323 | |  | |  | |  | |
| Yes | 913 | | | **97.4** | | 24 | | **2.6** | | 937 | | 1.03 | | 0.57-1.85 | |  | |  | |  | |  | |
| Fever (≥38°C or history of fever) |  | | |  | |  | |  | |  | |  | |  | |  | |  | |  | |  | |
| No | 336 | | | **97.4** | | 9 | | **2.6** | | 345 | | 1 | |  | | 0.3603 | |  | |  | |  | |
| Yes | 1654 | | | **97.8** | | 38 | | **2.3** | | 1692 | | 0.69 | | 0.31-1.50 | |  | |  | |  | |  | |
| Fever (≥39°C) |  | | |  | |  | |  | |  | |  | |  | |  | |  | |  | |  | |
| No | 1716 | | | **98.0** | | 35 | | **2.0** | | 1751 | | 1 | |  | | 0.1110 | |  | |  | |  | |
| Yes | 273 | | | **95.8** | | 12 | | **4.2** | | 285 | | 1.81 | | 0.90-3.63 | |  | |  | |  | |  | |
| Cough (observed) |  | | |  | |  | |  | |  | |  | |  | |  | |  | |  | |  | |
| No | 483 | | | **97.0** | | 15 | | **3.0** | | 498 | | 1 | |  | | 0.9307 | |  | |  | |  | |
| Yes | 1502 | | | **97.9** | | 32 | | **2.1** | | 1534 | | 0.97 | | 0.50-1.88 | |  | |  | |  | |  | |
| Wheeze (on auscultation) |  | | |  | |  | |  | |  | |  | |  | |  | |  | |  | |  | |
| No | 1049 | | | **96.5** | | 38 | | **3.5** | | 1087 | | 1 | |  | | 0.0745 | |  | |  | |  | |
| Yes | 931 | | | **99.0** | | 9 | | **1.0** | | 940 | | 0.49 | | 0.21-1.13 | |  | |  | |  | |  | |
| Grunting (observed) |  | | |  | |  | |  | |  | |  | |  | |  | |  | |  | |  | |
| No | 1761 | | | **98.1** | | 35 | | **2.0** | | 1796 | | 1 | |  | | 0.5396 | |  | |  | |  | |
| Yes | 222 | | | **94.9** | | 12 | | **5.1** | | 234 | | 0.77 | | 0.33-1.77 | |  | |  | |  | |  | |
| Crackling/ crepitations (on auscultation) |  | | |  | |  | |  | |  | |  | |  | |  | |  | |  | |  | |
| No | 555 | | | **96.9** | | 18 | | **3.1** | | 573 | | 1 | |  | | 0.8400 | |  | |  | |  | |
| Yes | 1427 | | | **98.0** | | 29 | | **2.0** | | 1456 | | 0.94 | | 0.50-1.75 | |  | |  | |  | |  | |
| Nasal flaring |  | | |  | |  | |  | |  | |  | |  | |  | |  | |  | |  | |
| No | 933 | | | **99.2** | | 8 | | **0.9** | | 941 | | 1 | |  | | 0.0157 | | 1 | |  | |  | |
| Yes | 1052 | | | **96.4** | | 39 | | **3.6** | | 1091 | | 2.63 | | 1.12-6.16 | |  | | 2.39 | | 1.00-5.73 | | 0.0375 | |
| Deep Breathing |  | | |  | |  | |  | |  | |  | |  | |  | |  | |  | |  | |
| No | 1410 | | | **97.6** | | 35 | | **2.4** | | 1445 | | 1 | |  | | 0.1672 | |  | |  | |  | |
| Yes | 575 | | | **98.0** | | 12 | | **2.0** | | 587 | | 1.79 | | 0.81-3.95 | |  | |  | |  | |  | |
| Skin Turgor |  | | |  | |  | |  | |  | |  | |  | |  | |  | |  | |  | |
| Normal | 1937 | | | **98.0** | | 40 | | **2.0** | | 1977 | | 1 | |  | | 0.0144 | |  | |  | |  | |
| Reduced | 47 | | | **87.0** | | 7 | | **13.0** | | 54 | | 3.51 | | 1.41-8.75 | |  | |  | |  | |  | |
| Capillary refill time (2) |  | | |  | |  | |  | |  | |  | |  | |  | |  | |  | |  | |
| <2 secs | 1057 | | | **99.1** | | 10 | | **0.9** | | 1067 | | 1 | |  | | 0.0506 | |  | |  | |  | |
| ≥ 2 secs | 760 | | | **96.1** | | 31 | | **3.9** | | 791 | | 2.46 | | 0.99-6.07 | |  | |  | |  | |  | |
| **Diagnostics** |  | | |  | |  | |  | |  | |  | |  | |  | |  | |  | |  | |
| Chest Radiograph |  | | |  | |  | |  | |  | |  | |  | |  | |  | |  | |  | |
| Abnormal (consolidation/ infiltrate) | 953 | | | **9.2** | | 27 | | **2.7** | | 980 | | 2.25 | | 1.07-4.72 | |  | |  | |  | |  | |
| Normal | 854 | | | **98.8** | | 10 | | **1.2** | | 864 | | 1 | |  | | 0.0118 | |  | |  | |  | |
| Uninterpretable | 143 | | | **94.7** | | 8 | | **5.3** | | 151 | | 4.07 | | 1.53-10.9 | |  | |  | |  | |  | |
| Leukocytosis |  | | |  | |  | |  | |  | |  | |  | |  | |  | |  | |  | |
| No | 969 | | | **97.0** | | 30 | | **3.0** | | 999 | | 1 | |  | | 0.3009 | |  | |  | |  | |
| Yes | 861 | | | **98.3** | | 15 | | **1.7** | | 876 | | 0.71 | | 0.37-1.36 | |  | |  | |  | |  | |
| Anaemia/ Hb level^8^ |  | | |  | |  | |  | |  | |  | |  | |  | |  | |  | |  | |
| None (≥ 9.3 g/dl) | 1422 | | | **98.1** | | 27 | | **1.9** | | 1449 | | 1 | |  | | 0.1816 | |  | |  | |  | |
| Moderate (6-9.2) | 537 | | | **96.9** | | 17 | | **3.1** | | 554 | | 1.52 | | 0.79-2.93 | |  | |  | |  | |  | |
| Severe (<6 g/dl) | 31 | | | **91.2** | | 3 | | **8.8** | | 34 | | 3.15 | | 0.86-11.5 | |  | |  | |  | |  | |
| Anaemia (2) |  | | |  | |  | |  | |  | |  | |  | |  | |  | |  | |  | |
| None | 1422 | | | **98.1** | | 27 | | **1.9** | | 1449 | | 1 | |  | | 0.1225 | |  | |  | |  | |
| Moderate-severe | 568 | | | **96.6** | | 20 | | **3.4** | | 588 | | 1.65 | | 0.88-3.09 | |  | |  | |  | |  | |
| C-reactive protein |  | | |  | |  | |  | |  | |  | |  | |  | |  | |  | |  | |
| 0-40 mg/L  (~ 80% viral infections) | 1281 | | | **97.7** | | 30 | | **2.3** | | 1311 | | 1 | |  | | 0.9232 | |  | |  | |  | |
| >40 mg/L  (~ 80% bacterial infections) | 387 | | | **97.0** | | 12 | | **3.0** | | 399 | | 1.03 | | 0.52-2.08 | |  | |  | |  | |  | |
|  |  | | |  | |  | |  | |  | |  | |  | |  | |  | |  | |  | |

^1^ A total of 17 unique factors were associated in univariable analyses (p<0.2) and did not suffer from sparse data (<5 events in a stratum),

if more than one categorisation of a variable was associated with mortality, the variable with fewer sub-strata was prioritised for inclusion into the final multivariable model due to the small number of events. Model building using backwards stepwise regression began with the 9 factors associated in univariable analyses (p<0.05), the model was reduced to include only those associated (p<0.05) in multivariable analysis. The other factors associated (p<0.2) in univariable analyses were then added in iteratively to check their associations in the final model at the end, none were associated. When restricted to data from non-Zambian sites, age and HIV exposure were no longer significant factors in the multivariable analysis, hypoxaemia and anthropometry remained highly significant risk factors for poor outcome, history of diarrhoea and nasal flaring became significant risk factors. Due to the low number of events there was limited power to detect other associations

**S5 Table. Performance of the multivariable model in 2-59 month olds by country and age group**

| **Site** | **Performance statistic (AUC) of the multivariable model in 2-59 month olds** |
| --- | --- |
|  |  |
|  |  |
| **All** | 0.81 |
| **Country^1^** |  |
| Kenya | 0.83 |
| Gambia | 0.69 |
| Mali | 0.86 |
| Zambia | 0.77 |
| South Africa | 0.76 |
| Thailand | 0.60 |
| Bangladesh | 0.71 |
| **Age** |  |
| 2-11 months | 0.77 |
| 12-59 months | 0.85 |

**S6 Table. The risk of death among different groups of LCWI pneumonia cases who presented to hospital, were HIV negative and aged 2-59 months**

| **Scenario** | **Population risk groups** | **Died** | | | | | **Percentage of LCWI cases^1^** | **Percentage of deaths in risk group** |
| --- | --- | --- | --- | --- | --- | --- | --- | --- |
|  |  | **No** | | **Yes** | | **Total** |  |  |
|  |  | **n** | **%** | **n** | **%** | **n** |  |  |
|  | **All LCWI pneumonia cases** | 2113 | **96.5** | 76 | **3.5** | 2189 | 100% | 100% |
| **A** | 1. **Infants** | 1208 | **95.3** | 59 | **4.7** | 1267 | 57.9% | 78% |
|  | **Comparison group 1 (non infants)** | 905 | **98.2** | 17 | **1.8** | 922 |  |  |
|  | 1. **Infants + Hypoxaemic** | 339 | **91.4** | 32 | **8.6** | 371 | 16.9% | 42% |
|  | **Comparison group 2** | 1774 | **97.6** | 44 | **2.4** | 1818 |  |  |
|  | 1. **Infants + low MUAC-WAZ** | 209 | **87.8** | 29 | **12.2** | 238 | 10.9% | 38% |
|  | **Comparison group 3** | 1904 | **97.6** | 47 | **2.4** | 1951 |  |  |
|  | 1. **Infants + HIV exposed** | 134 | **88.7** | 17 | **11.3** | 151 | 6.9% | 22% |
|  | **Comparison group 4** | 1979 | **97.1** | 59 | **2.9** | 2038 |  |  |
|  | 1. **Infants + Hypoxaemic + low MUAC-WAZ** | 66 | **79.5** | 17 | **20.5** | 83 | 3.8% | 22% |
|  | **Comparison group 5** | 2047 | **97.2** | 59 | **2.8** | 2106 |  |  |
|  | 1. **Infants + Hypoxaemic + HIV exposed** | 77 | **89.5** | 9 | **10.5** | 86 | 3.9% | 12% |
|  | **Comparison group 6** | 2036 | **96.8** | 67 | **3.2** | 2103 |  |  |
|  | 1. **Infants + HIV exposed + low MUAC-WAZ** | 26 | **86.7** | 4 | **13.3** | 30 | 1.4% | 5% |
|  | **Comparison group 7** | 2087 | **96.7** | 72 | **3.3** | 2159 |  |  |
|  | 1. **Hypoxaemic (<92%) + HIV exposed + low MUAC-WAZ infants** | 13 | **86.7** | 2 | **13.3** | 15 | 0.7% | 3% |
|  | **Comparison group 8** | 2100 | **96.6** | 74 | **3.4** | 2174 |  |  |
| **B** | 1. **Hypoxaemic (<92%)** | 536 | **92.7** | 42 | **7.3** | 578 | 26.4% | 55% |
|  | **Comparison group 9** | 1577 | **97.9** | 34 | **2.1** | 1611 |  |  |
|  | 1. **Hypoxaemic (<92%) + low MUAC-WAZ** | 96 | **80.0** | 24 | **20.0** | 120 | 5.5% | 32% |
|  | **Comparison group 10** | 2017 | **97.5** | 52 | **2.5** | 2069 |  |  |
|  | 1. **Hypoxaemic (<92%) + HIV exposed** | 105 | **92.1** | 9 | **7.9** | 114 | 5.2% | 12% |
|  | **Comparison group 11** | 2008 | **96.8** | 67 | **3.2** | 2075 |  |  |
|  | 1. **Hypoxaemic (<92%) + HIV exposed + low MUAC-WAZ** | 17 | **89.5** | 2 | **10.5** | 19 | 0.9% | 3% |
|  | **Comparison group 12** | 2096 | **96.6** | 74 | **3.4** | 2170 |  |  |
| **C** | 1. **Low MUAC-WAZ** | 304 | **88.4** | 40 | **11.6** | 344 | 15.7% | 53% |
|  | **Comparison group 13** | 1809 | **98.1** | 36 | **2.0** | 1845 |  |  |
|  | 1. **Low-MUAC-WAZ + HIV exposed** | 32 | **88.9** | 4 | **11.1** | 36 | 1.6% | 5% |
|  | **Comparison group 14** | 2081 | **96.7** | 72 | **3.3** | 2153 |  |  |
| **D** | 1. **HIV exposed** | 183 | **91.0** | 18 | **9.0** | 201 | 9.2% | 24% |
|  | **Comparison group 15** | 1930 | **97.1** | 58 | **2.9** | 1988 |  |  |
|  | 1. **Infants or Hypoxaemic** | 1405 | **95.3** | 69 | **4.7** | 1474 | 67% | 91% |
|  | **Comparison group 16** | 708 | **99.0** | 7 | **1.0** | 715 |  |  |
|  | 1. **Infants or low MUAC-WAZ** | 1303 | **94.9** | 70 | **5.1** | 1373 | 63% | 92% |
|  | **Comparison group 17** | 810 | **99.3** | 6 | **0.7** | 816 |  |  |
|  | 1. **Infants or HIV exposed** | 1257 | **95.4** | 60 | **4.6** | 1317 | 60% | 79% |
|  | **Comparison group 18** | 856 | **98.2** | 16 | **1.8** | 872 |  |  |
|  | 1. **Infants or Hypoxaemic or low MUAC-WAZ** | 1470 | **95.3** | 73 | **4.7** | 1543 | 70% | 96% |
|  | **Comparison group 19** | 643 | **99.5** | 3 | **0.5** | 646 |  |  |
|  | 1. **Infants or Hypoxaemic or HIV exposed** | 1426 | **95.3** | 70 | **4.7** | 1496 | 68% | 92% |
|  | **Comparison group 20** | 687 | **99.1** | 6 | **0.9** | 693 |  |  |
|  | 1. **Infants or HIV exposed or low MUAC-WAZ** | 1346 | **95.5** | 71 | **5.0** | 1417 | 65% | 93% |
|  | **Comparison group 21** | 767 | **99.4** | 5 | **0.7** | 772 |  |  |
|  | 1. **Hypoxaemic (<92%), or HIV exposed, or low MUAC-WAZ or infants** | 1489 | **95.3** | 74 | **4.7** | 1563 | 71% | 97% |
|  | **Comparison group 22** | 624 | **99.7** | 2 | **0.3** | 626 |  |  |
| **E** | 1. **Hypoxaemic (<92%), or low MUAC-WAZ** | 744 | **92.8** | 58 | **7.2** | 802 | 37% | 76% |
|  | **Comparison group 23** | 1369 | **98.7** | 18 | **1.3** | 1387 |  |  |
| **F** | 1. **Hypoxaemic (<92%), or HIV exposed** | 614 | **92.3** | 51 | **7.7** | 665 | 30% | 67% |
|  | **Comparison group 24** | 1499 | **98.4** | 25 | **1.6** | 1524 |  |  |
| **G** | 1. **Hypoxaemic (<92%), or HIV exposed, or low MUAC-WAZ** | 807 | **92.6** | 65 | **7.5** | 872 | 40% | 86% |
|  | **Comparison group 25** | 1306 | **99.2** | 11 | **0.8** | 1317 |  |  |
| **H** | 1. **Low-MUAC-WAZ, or HIV exposed** | 455 | **89.4** | 54 | **10.6** | 509 | 23% | 71% |
|  | **Comparison group 26** | 1658 | **98.7** | 22 | **1.3** | 1680 |  |  |
| **I** | **LCWI cases with underlying conditions^2^** | 481 | **90.6** | 50 | **9.4** | 531 | 24.3% | 66% |
|  | **No underlying conditions** | 1632 | **98.4** | 26 | **1.6** | 1658 |  |  |

^1^ Proportion of LCWI pneumonia cases presenting to hospital in the identified high risk group and the proportion of deaths that occurred in the identified high risk group. NB. Comparison groups include those with missing data on the risk factor identified.

^2^ Any underlying condition was defined as: Severe malnutrition (WFH z-score <-3 regardless of age, or MUAC <115 mm for children ≥ 6 months, or pedal edema on admission, or admission diagnosis of Kwashiorkor), or HIV exposure, or premature and less than 6 months of age, or a diagnosis at admission of: heart disease, including congenital heart conditions; developmental delays, including cerebral palsy; congenital abnormalities (e.g. Trisomy 21, G6PD).

Low MUAC-WAZ is low or very low MUAC and low or very low WAZ z-scores if <3 months of age.

Interpretation:

Scenario A: If all infants with LCWI pneumonia were admitted, but children who were above 1 year of age were treated at home: 58% of all LCWI pneumonia cases would have been admitted to hospital, including 78% of the deaths.

Scenario B: If all hypoxaemic cases (<92% saturation or on oxygen at admission) were admitted but non-hypoxaemic LCWI cases were treated at home: 26% of all LCWI cases would have been admitted, including 55% of the deaths.

Scenario C: If all children with malnutrition (low or very low mid-upper-arm circumference or weight-for-age scores) were admitted to hospital but well-nourished children were treated at home: 16% of the PERCH LCWI cases would have been admitted, including 53% of the deaths.

Scenario D: If all HIV exposed LCWI cases were admitted but unexposed children were treated at home: 9% of LCWI cases would have been admitted, including24% of the deaths.

Scenario E: If LCWI pneumonia cases with hypoxaemia or low MUAC/WAZ were admitted: 37% of LCWI cases would have been admitted, including 76% of the deaths.

Scenario F: If LCWI pneumonia cases with hypoxaemia or HIV-exposure were admitted: 30% of LCWI cases would have been admitted, including 67% of the deaths.

Scenario G: If LCWI pneumonia cases with hypoxaemia, or HIV exposure, or low MUAC/WAZ were admitted: 40% of the LCWI cases would have been admitted, including 86% of the deaths.

Scenario H: If LCWI pneumonia cases with low MUAC/WAZ or those HIV exposed were admitted: 23% of all LCWI cases would have been admitted, including 71% of the deaths.

Scenario I (top): If LCWI pneumonia cases with any ‘underlying condition’ as per IMCI guidelines were admitted i.e. those severely malnourished (WFH z-score <-3 regardless of age, or MUAC <115 mm for children ≥ 6 months, or pedal oedema on admission, or admission diagnosis of kwashiorkor), or children who were HIV exposed or premature and <6 months of age or with a diagnosis on admission of: heart disease, developmental delays, or congenital abnormalities (e.g. Trisomy 21, glucose-6-phosphate dehydrogenase (G6PD) deficiency): 24% of all LCWI cases would have been admitted, including 66% of the deaths.

**S7 Table. The prevalence of comorbidities and underlying conditions in HIV-negative LCWI pneumonia cases aged 2-59 months**

| **Characteristic** |  | **Died** | | | | | | | **Unadjusted** | | | | | | | |
| --- | --- | --- | --- | --- | --- | --- | --- | --- | --- | --- | --- | --- | --- | --- | --- | --- |
|  |  | **No** | **Yes** | | | **Total** | | **OR** | | | **95%CI** | | **LRT**  **p-value^1^** | | |  |
|  |  | **n** | **%** | **n** | **%** | | **n** | | |  | |  | |  |  |  |
| **All LCWI pneumonia cases** |  | 2113 | **96.5** | 76 | **3.5** | | 2189 | | |  | |  | |  |  |  |
| Severe malnutrition^1^ | No | 1880 | **97.8** | 43 | **2.2** | | 1923 | | | 1 | |  | |  |  |  |
|  | Yes | 233 | **87.6** | 33 | **12.5** | | 266 | | | 5.03 | | 2.99-8.45 | | <0.0001 |  |  |
| HIV exposure | Unexposed | 1716 | **97.2** | 50 | **2.8** | | 1766 | | | 1 | |  | | 0.0096 |  |  |
|  | Exposed | 183 | **97.2** | 18 | **9.0** | | 201 | | | 2.63 | | 1.27-5.43 | |  |  |  |
| Premature & <6months | No | 2024 | 96.8 | 68 | 3.3 | | 2092 | | | 1 | |  | |  |  |  |
|  | Yes | 89 | 91.8 | 8 | 8.3 | | 97 | | | 2.58 | | 1.13-5.89 | | 0.038 |  |  |
| Underlying conditions^2^ | No | 2056 | **96.9** | 65 | **3.1** | | 2121 | | | 1 | |  | |  |  |  |
|  | Yes | 57 | **83.8** | 11 | **16.2** | | 68 | | | 6.62 | | 3.06-14.3 | | <0.0001 |  |  |
| **Any of the above** | No | 1632 | **98.4** | 26 | **1.6** | | 1658 | | | 1 | |  | |  |  |  |
|  | Yes | 481 | **90.6** | 50 | **9.4** | | 531 | | | 5.01 | | 2.98-8.42 | | <0.0001 |  |  |
|  |  |  |  |  |  | |  | | |  | |  | |  |  |  |

^1^ Severe malnutrition defined as: WFH z-score <-3 regardless of age, or MUAC <115 mm for children ≥ 6 months, or pedal edema on admission, or admission diagnosis of Kwashiorkor.

^2^ Other underlying conditions were defined if recognised at admission as: heart disease, including congenital heart conditions; developmental delays, including cerebral palsy; congenital abnormalities (e.g. Trisomy 21, G6PD).

These factors were not used to restrict the analysis dataset as although LCWI pneumonia cases with any underlying condition or comorbidity are recommended for admission to hospital in IMCI guidelines – the proportion of these cases who are correctly identified at admission is unknown.

**S8 Table.** **Factors associated with mortality after presentation to hospital with LCWI pneumonia in HIV-negative children aged 2-11 months**

| **Characteristic** | **Died** | | | | | | | | | | | **Unadjusted** | | | | | | **Adjusted** | | | | | |
| --- | --- | --- | --- | --- | --- | --- | --- | --- | --- | --- | --- | --- | --- | --- | --- | --- | --- | --- | --- | --- | --- | --- | --- |
|  | **No** | | | | | **Yes** | | | | **Total** | | **OR^1^** | | **95%CI** | | **LRT**  **p-value^1^** | | **aOR** | | **95%CI** | | **LRT**  **p-value^1^** | |
|  | **n** | | | **%** | | **n** | | **%** | | **n** | |  | |  | |  |  |  | |  | |  |  |
| **All** | 1208 | | | **95.3** | | 59 | | **4.7** | | 1267 | |  | |  | |  | |  | |  | |  | |
| **Country^1^** |  | | |  | |  | |  | |  | |  | |  | |  | |  | |  | |  | |
| Kenya | 150 | | | **97.4** | | 4 | | **2.6** | | 154 | |  | |  | |  | |  | |  | |  | |
| Gambia | 295 | | | **98.7** | | 4 | | **1.3** | | 299 | |  | |  | |  | |  | |  | |  | |
| Mali | 154 | | | **90.6** | | 16 | | **9.4** | | 170 | |  | |  | |  | |  | |  | |  | |
| Zambia | 91 | | | **78.5** | | 25 | | **21.6** | | 116 | |  | |  | |  | |  | |  | |  | |
| South Africa | 271 | | | **97.8** | | 6 | | **2.2** | | 277 | |  | |  | |  | |  | |  | |  | |
| Thailand | 51 | | | **96.2** | | 2 | | **3.8** | | 53 | |  | |  | |  | |  | |  | |  | |
| Bangladesh | 196 | | | **99.0** | | 2 | | **1.01** | | 198 | |  | |  | |  | |  | |  | |  | |
| **Age** |  | | |  | |  | |  | |  | |  | |  | |  | |  | |  | |  | |
| 2-5 months | 663 | | | **94.4** | | 39 | | **5.6** | | 702 | | 1.44 | | 0.81-2.55 | | 0.2105 | |  | |  | |  | |
| 6-11 months | 545 | | | **96.5** | | 20 | | **3.5** | | 565 | | 1 | |  | |  | |  | |  | |  | |
| **Sex** |  | | |  | |  | |  | |  | |  | |  | |  | |  | |  | |  | |
| Male | 756 | | | **96.7** | | 26 | | **3.3** | | 782 | | 1 | |  | |  | |  | |  | |  | |
| Female | 452 | | | **93.2** | | 33 | | **6.8** | | 485 | | 1.92 | | 1.11-3.32 | | 0.0200 | |  | |  | |  | |
| **Medical History** |  | | |  | |  | |  | |  | |  | |  | |  | |  | |  | |  | |
| History of difficulty breathing |  | | |  | |  | |  | |  | |  | |  | |  | |  | |  | |  | |
| No | 35 | | | **92.1** | | 3 | | **7.9** | | 38 | | 1 | |  | | 0.5112 | |  | |  | |  | |
| Yes | 1172 | | | **95.4** | | 56 | | **4.6** | | 1228 | | 0.64 | | 0.18-2.28 | |  | |  | |  | |  | |
| History of cough |  | | |  | |  | |  | |  | |  | |  | |  | |  | |  | |  | |
| No | 20 | | | **80.0** | | 5 | | **20.0** | | 25 | | 1 | |  | | 0.0375 | |  | |  | |  | |
| Yes | 1188 | | | **95.7** | | 54 | | **4.4** | | 1242 | | 0.28 | | 0.09-0.84 | |  | |  | |  | |  | |
| History of inability to feed |  | | |  | |  | |  | |  | |  | |  | |  | |  | |  | |  | |
| No | 1163 | | | **95.5** | | 55 | | **4.5** | | 1218 | | 1 | |  | | 0.1607 | |  | |  | |  | |
| Yes | 45 | | | **91.8** | | 4 | | **8.2** | | 49 | | 2.45 | | 0.77-7.82 | |  | |  | |  | |  | |
| History of ear discharge |  | | |  | |  | |  | |  | |  | |  | |  | |  | |  | |  | |
| No | 1203 | | | **95.4** | | 58 | | **4.6** | | 1261 | | 1 | |  | | 0.1007 | |  | |  | |  | |
| Yes | 5 | | | **83.3** | | 1 | | **16.7** | | 6 | | 10.9 | | 1.12-106 | |  | |  | |  | |  | |
| History of diarrhea |  | | |  | |  | |  | |  | |  | |  | |  | |  | |  | |  | |
| No | 1047 | | | **96.4** | | 39 | | **3.6** | | 1086 | | 1 | |  | | 0.0088 | | 1 | |  | |  | |
| Yes | 161 | | | **89.0** | | 20 | | **11.1** | | 181 | | 2.31 | | 1.26-4.23 | |  | | 2.40 | | 1.24-4.64 | | 0.0114 | |
| Maximum duration of illness^6^ | | |  | |  | |  | |  | |  | |  | |  | |  | |  | |  | |  |
| 0-2 days | 369 | | | **96.6** | | 13 | | **3.4** | | 382 | | 1 | |  | | 0.4864 | |  | |  | |  | |
| 3-5 days | 613 | | | **95.8** | | 27 | | **4.2** | | 640 | | 1.33 | | 0.66-2.70 | |  | |  | |  | |  | |
| >5 days | 221 | | | **92.5** | | 18 | | **7.5** | | 239 | | 1.60 | | 0.74-3.48 | |  | |  | |  | |  | |
| HIV exposure |  | | |  | |  | |  | |  | |  | |  | |  | |  | |  | |  | |
| Unexposed | 930 | | | **96.4** | | 35 | | **3.6** | | 965 | | 1 | |  | | 0.0082 | |  | |  | |  | |
| Exposed | 134 | | | **88.7** | | 17 | | **11.3** | | 151 | | 2.84 | | 1.32-6.13 | |  | |  | |  | |  | |
| **Vaccination status** |  | | |  | |  | |  | |  | |  | |  | |  | |  | |  | |  | |
| DTP vaccination status |  | | |  | |  | |  | |  | |  | |  | |  | |  | |  | |  | |
| None | 136 | | | **95.1** | | 7 | | **4.9** | | 143 | | 1 | |  | | 0.3106 | |  | |  | |  | |
| Partial | 205 | | | **97.2** | | 6 | | **2.8** | | 211 | | 0.41 | | 0.13-1.29 | |  | |  | |  | |  | |
| Full | 842 | | | **94.9** | | 45 | | **5.1** | | 887 | | 0.63 | | 0.26-1.51 | |  | |  | |  | |  | |
| Hib vaccination |  | | |  | |  | |  | |  | |  | |  | |  | |  | |  | |  | |
| None | 176 | | | **96.2** | | 7 | | **3.8** | | 183 | | 1 | |  | | 0.5023 | |  | |  | |  | |
| Partial | 171 | | | **97.7** | | 4 | | **2.3** | | 175 | | 0.51 | | 0.13-1.94 | |  | |  | |  | |  | |
| Full | 836 | | | **94.7** | | 47 | | **5.3** | | 883 | | 0.90 | | 0.34-2.33 | |  | |  | |  | |  | |
| PCV vaccination |  | | |  | |  | |  | |  | |  | |  | |  | |  | |  | |  | |
| None | 449 | | | **93.2** | | 33 | | **6.9** | | 482 | | 1 | |  | | 0.5073 | |  | |  | |  | |
| Partial | 146 | | | **98.0** | | 3 | | **2.0** | | 149 | | 0.45 | | 0.11-1.83 | |  | |  | |  | |  | |
| Full | 591 | | | **96.4** | | 22 | | **3.6** | | 613 | | 0.71 | | 0.28-1.75 | |  | |  | |  | |  | |
| **Birth history/ Anthropometrics** | |  | |  | |  | |  | |  | |  | |  | |  | |  | |  | |  | |
| Weight for height^7^ |  | | |  | |  | |  | |  | |  | |  | |  | |  | |  | |  | |
| Very low | 119 | | | **83.8** | | 23 | | **16.2** | | 142 | | 6.71 | | 3.48-12.9 | | <0.0001 | |  | |  | |  | |
| Low | 144 | | | **92.9** | | 11 | | **7.1** | | 155 | | 3.51 | | 1.61-7.63 | |  | |  | |  | |  | |
| Normal-high | 916 | | | **97.6** | | 23 | | **2.5** | | 939 | | 1 | |  | |  | |  | |  | |  | |
| Weight for age^7^ |  | | |  | |  | |  | |  | |  | |  | |  | |  | |  | |  | |
| Very low | 184 | | | **88.5** | | 24 | | **11.5** | | 208 | | 5.68 | | 2.91-11.1 | | <0.0001 | |  | |  | |  | |
| Low | 187 | | | **91.7** | | 17 | | **8.3** | | 204 | | 4.08 | | 2.00-8.35 | |  | |  | |  | |  | |
| Normal-high | 832 | | | **97.9** | | 18 | | **2.1** | | 850 | | 1 | |  | |  | |  | |  | |  | |
| Height for age^7^ |  | | |  | |  | |  | |  | |  | |  | |  | |  | |  | |  | |
| Very low | 186 | | | **90.3** | | 20 | | **9.7** | | 206 | | 1.97 | | 1.03-3.77 | | 0.1325 | |  | |  | |  | |
| Low | 141 | | | **95.3** | | 7 | | **4.7** | | 148 | | 1.12 | | 0.47-2.68 | |  | |  | |  | |  | |
| Normal-high | 860 | | | **96.5** | | 31 | | **3.5** | | 891 | | 1 | |  | |  | |  | |  | |  | |
| MUAC for age^7^ |  | | |  | |  | |  | |  | |  | |  | |  | |  | |  | |  | |
| Very low | 66 | | | **84.6** | | 12 | | **15.4** | | 78 | | 4.95 | | 2.16-11.4 | | 0.0007 | |  | |  | |  | |
| Low | 79 | | | **90.8** | | 8 | | **9.2** | | 87 | | 2.51 | | 1.04-6.06 | |  | |  | |  | |  | |
| Normal-high | 808 | | | **96.9** | | 26 | | **3.1** | | 834 | | 1 | |  | |  | |  | |  | |  | |
| MUAC/ Weight for height if <3months^7^ |  | | |  | |  | |  | |  | |  | |  | |  | |  | |  | |  | |
| Very low | 85 | | | **85.9** | | 14 | | **14.1** | | 99 | | 4.43 | | 2.13-9.23 | | 0.0003 | |  | |  | |  | |
| Low | 101 | | | **91.0** | | 10 | | **9.0** | | 111 | | 2.42 | | 1.11-5.29 | |  | |  | |  | |  | |
| Normal-high | 974 | | | **96.7** | | 33 | | **3.3** | | 1007 | | 1 | |  | |  | |  | |  | |  | |
| MUAC/ Weight for age if <3months^7^ |  | | |  | |  | |  | |  | |  | |  | |  | |  | |  | |  | |
| Very low | 101 | | | **87.1** | | 15 | | **12.9** | | 116 | | 4.74 | | 2.28-9.84 | | <0.0001 | | 3.69 | | 1.71-7.93 | | 0.0003 | |
| Low | 108 | | | **88.5** | | 14 | | **11.5** | | 122 | | 3.85 | | 1.87-7.90 | |  | | 3.42 | | 1.63-7.19 | |  | |
| Normal-high | 958 | | | **97.2** | | 28 | | **2.8** | | 986 | | 1 | |  | |  | | 1 | |  | |  | |
| BMI for age^7^ |  | | |  | |  | |  | |  | |  | |  | |  | |  | |  | |  | |
| Very low | 144 | | | **85.2** | | 25 | | **14.8** | | 169 | | 5.97 | | 3.15-11.3 | | <0.0001 | |  | |  | |  | |
| Low | 149 | | | **94.3** | | 9 | | **5.7** | | 158 | | 2.74 | | 1.20-6.24 | |  | |  | |  | |  | |
| Normal-high | 892 | | | **97.4** | | 24 | | **2.6** | | 916 | | 1 | |  | |  | |  | |  | |  | |
| Premature or small at birth |  | | |  | |  | |  | |  | |  | |  | |  | |  | |  | |  | |
| No | 888 | | | **96.3** | | 34 | | **3.7** | | 922 | | 1 | |  | | 0.0096 | |  | |  | |  | |
| Yes | 311 | | | **93.1** | | 23 | | **6.9** | | 334 | | 2.19 | | 1.23-3.91 | |  | |  | |  | |  | |
| Birthweight |  | | |  | |  | |  | |  | |  | |  | |  | |  | |  | |  | |
| Normal-high (>2490g) | 929 | | | **96.3** | | 36 | | **3.7** | | 965 | | 1 | |  | | 0.0089 | |  | |  | |  | |
| Low (≤ 2490g) | 266 | | | **92.7** | | 21 | | **7.3** | | 287 | | 2.25 | | 1.25-4.05 | |  | |  | |  | |  | |
| **Signs on clinical exam.** | | |  | |  | |  | |  | |  | |  | |  | |  | |  | |  | |  |
| O2 saturation (60, 92) |  | | |  | |  | |  | |  | |  | |  | |  | |  | |  | |  | |
| <60% | 5 | | | **83.3** | | 1 | | **16.7** | | 6 | | 4.44 | | 0.36-54.3 | | 0.0006 | |  | |  | |  | |
| 60-91%^2^ | 334 | | | **91.5** | | 31 | | **8.5** | | 365 | | 3.19 | | 1.74-5.84 | |  | |  | |  | |  | |
| ≥ 92% (or 90% in SA/ZAM) | 868 | | | **97.0** | | 27 | | **3.0** | | 895 | | 1 | |  | |  | |  | |  | |  | |
| O2 saturation (80, 92) |  | | |  | |  | |  | |  | |  | |  | |  | |  | |  | |  | |
| <80% | 48 | | | **80.0** | | 12 | | **20.0** | | 60 | | 7.31 | | 3.12-17.1 | | <0.0001 | | 6.84 | | 2.78-16.8 | | 0.0002 | |
| 80-91%^2^ | 291 | | | **93.6** | | 20 | | **6.4** | | 311 | | 2.41 | | 1.24-4.66 | |  | | 2.14 | | 1.07-4.29 | |  | |
| ≥ 92% or ≥ 90% in SA/ ZAM | 866 | | | **97.0** | | 27 | | **3.0** | | 893 | | 1 | |  | |  | | 1 | |  | |  | |
| Hypoxaemia |  | | |  | |  | |  | |  | |  | |  | |  | |  | |  | |  | |
| No (≥ 90%) | 908 | | | **96.8** | | 30 | | **3.2** | | 938 | | 1 | |  | | 0.0002 | |  | |  | |  | |
| Yes (<90%)^3^ | 299 | | | **91.2** | | 29 | | **8.8** | | 328 | | 3.19 | | 1.75-5.84 | |  | |  | |  | |  | |
| Hypoxaemia |  | | |  | |  | |  | |  | |  | |  | |  | |  | |  | |  | |
| No | 866 | | | **97.0** | | 27 | | **3.0** | | 893 | | 1 | |  | | 0.0001 | |  | |  | |  | |
| Yes (<92%, or <90% in SA and ZAM)^4^ | 339 | | | **91.4** | | 32 | | **8.6** | | 371 | | 3.22 | | 1.76-5.87 | |  | |  | |  | |  | |
| Elevated respiratory rate for age^5^ |  | | |  | |  | |  | |  | |  | |  | |  | |  | |  | |  | |
| No | 197 | | | **95.6** | | 9 | | **4.4** | | 206 | | 1 | |  | | 0.9372 | |  | |  | |  | |
| Yes | 1005 | | | **95.4** | | 49 | | **4.7** | | 1054 | | 0.97 | | 0.45-2.08 | |  | |  | |  | |  | |
| Elevated respiratory rate (≥ 70 bpm) |  | | |  | |  | |  | |  | |  | |  | |  | |  | |  | |  | |
| No | 945 | | | **96.2** | | 37 | | **3.8** | | 982 | | 1 | |  | | 0.1526 | |  | |  | |  | |
| ≥ 70 bpm | 257 | | | **92.5** | | 21 | | **7.6** | | 278 | | 1.55 | | 0.86-2.79 | |  | |  | |  | |  | |
| Elevated heart rate for age^5^ |  | | |  | |  | |  | |  | |  | |  | |  | |  | |  | |  | |
| No | 654 | | | **95.8** | | 29 | | **4.3** | | 683 | | 1 | |  | | 0.9251 | |  | |  | |  | |
| Yes | 550 | | | **94.8** | | 30 | | **5.2** | | 580 | | 1.03 | | 0.59-1.78 | |  | |  | |  | |  | |
| Fever (≥ 38°C or history of fever) |  | | |  | |  | |  | |  | |  | |  | |  | |  | |  | |  | |
| No | 211 | | | **94.2** | | 13 | | **5.8** | | 224 | | 1 | |  | | 0.3023 | |  | |  | |  | |
| Yes | 997 | | | **95.6** | | 46 | | **4.4** | | 1043 | | 0.69 | | 0.35-1.37 | |  | |  | |  | |  | |
| Fever (≥ 39°C) |  | | |  | |  | |  | |  | |  | |  | |  | |  | |  | |  | |
| No | 1040 | | | **96.4** | | 39 | | **3.6** | | 1079 | | 1 | |  | | 0.0229 | |  | |  | |  | |
| Yes | 167 | | | **89.3** | | 20 | | **10.7** | | 187 | | 2.11 | | 1.13-3.95 | |  | |  | |  | |  | |
| Cough (observed) |  | | |  | |  | |  | |  | |  | |  | |  | |  | |  | |  | |
| No | 318 | | | **93.8** | | 21 | | **6.2** | | 339 | | 1 | |  | | 0.2184 | |  | |  | |  | |
| Yes | 887 | | | **95.9** | | 38 | | **4.1** | | 925 | | 0.68 | | 0.38-1.24 | |  | |  | |  | |  | |
| Wheeze (on auscultation) |  | | |  | |  | |  | |  | |  | |  | |  | |  | |  | |  | |
| No | 705 | | | **93.6** | | 48 | | **6.4** | | 753 | | 1 | |  | | 0.3079 | |  | |  | |  | |
| Yes | 495 | | | **97.8** | | 11 | | **2.2** | | 506 | | 0.67 | | 0.31-1.47 | |  | |  | |  | |  | |
| Grunting (observed) |  | | |  | |  | |  | |  | |  | |  | |  | |  | |  | |  | |
| No | 1038 | | | **96.3** | | 40 | | **3.7** | | 1078 | | 1 | |  | | 0.3518 | |  | |  | |  | |
| Yes | 164 | | | **89.6** | | 19 | | **10.4** | | 183 | | 1.38 | | 0.70-2.72 | |  | |  | |  | |  | |
| Crackling/ crepitations (on auscultation) |  | | |  | |  | |  | |  | |  | |  | |  | |  | |  | |  | |
| No | 362 | | | **93.1** | | 27 | | **6.9** | | 389 | | 1 | |  | | 0.3568 | |  | |  | |  | |
| Yes | 840 | | | **96.3** | | 32 | | **3.7** | | 872 | | 0.77 | | 0.44-1.35 | |  | |  | |  | |  | |
| Nasal flaring |  | | |  | |  | |  | |  | |  | |  | |  | |  | |  | |  | |
| No | 521 | | | **96.3** | | 20 | | **3.7** | | 541 | | 1 | |  | | 0.6008 | |  | |  | |  | |
| Yes | 683 | | | **94.6** | | 39 | | **5.4** | | 722 | | 1.18 | | 0.63-2.19 | |  | |  | |  | |  | |
| Deep Breathing |  | | |  | |  | |  | |  | |  | |  | |  | |  | |  | |  | |
| No | 932 | | | **94.7** | | 52 | | **5.3** | | 984 | | 1 | |  | | 0.8245 | |  | |  | |  | |
| Yes | 272 | | | **97.5** | | 7 | | **2.5** | | 279 | | 0.90 | | 0.35-2.31 | |  | |  | |  | |  | |
| Skin Turgor |  | | |  | |  | |  | |  | |  | |  | |  | |  | |  | |  | |
| Normal | 1172 | | | **95.9** | | 50 | | **4.1** | | 1222 | | 1 | |  | | 0.0009 | |  | |  | |  | |
| Reduced | 31 | | | **77.5** | | 9 | | **22.5** | | 40 | | 5.14 | | 2.12-12.4 | |  | |  | |  | |  | |
| Capillary refill time |  | | |  | |  | |  | |  | |  | |  | |  | |  | |  | |  | |
| <2 secs | 585 | | | **98.5** | | 9 | | **1.5** | | 594 | | 1 | |  | | 0.0369 | |  | |  | |  | |
| ≥ 2 secs | 499 | | | **93.1** | | 37 | | **6.9** | | 536 | | 2.64 | | 1.05-6.67 | |  | |  | |  | |  | |
| **Diagnostics** |  | | |  | |  | |  | |  | |  | |  | |  | |  | |  | |  | |
| Chest Radiograph |  | | |  | |  | |  | |  | |  | |  | |  | |  | |  | |  | |
| Abnormal (consolidation/ infiltrate) | 559 | | | **96.1** | | 23 | | **4.0** | | 582 | | 1.55 | | 0.77-3.13 | | 0.0408 | |  | |  | |  | |
| Normal | 504 | | | **97.5** | | 13 | | **2.5** | | 517 | | 1 | |  | |  | |  | |  | |  | |
| Uninterpretable | 111 | | | **90.2** | | 12 | | **9.8** | | 123 | | 3.04 | | 1.31-7.08 | |  | |  | |  | |  | |
| Leukocytosis |  | | |  | |  | |  | |  | |  | |  | |  | |  | |  | |  | |
| No | 680 | | | **95.1** | | 35 | | **4.9** | | 715 | | 1 | |  | | 0.6130 | |  | |  | |  | |
| Yes | 416 | | | **95.2** | | 21 | | **4.8** | | 437 | | 1.16 | | 0.65-2.10 | |  | |  | |  | |  | |
| Anaemia/ Hb level^8^ |  | | |  | |  | |  | |  | |  | |  | |  | |  | |  | |  | |
| None (≥ 9.3 g/dl) | 846 | | | **96.0** | | 35 | | **4.0** | | 881 | | 1 | |  | | 0.1982 | |  | |  | |  | |
| Moderate (6-9.2) | 341 | | | **93.9** | | 22 | | **6.1** | | 363 | | 1.72 | | 0.96-3.09 | |  | |  | |  | |  | |
| Severe (<6 g/dl) | 21 | | | **91.3** | | 2 | | **8.7** | | 23 | | 1.43 | | 0.30-6.80 | |  | |  | |  | |  | |
| Anaemia (2) |  | | |  | |  | |  | |  | |  | |  | |  | |  | |  | |  | |
| None | 846 | | | **96.0** | | 35 | | **4.0** | | 881 | | 1 | |  | | 0.0744 | |  | |  | |  | |
| Moderate-severe | 362 | | | **93.8** | | 24 | | **6.2** | | 386 | | 1.69 | | 0.96-2.99 | |  | |  | |  | |  | |
| C-reactive protein |  | | |  | |  | |  | |  | |  | |  | |  | |  | |  | |  | |
| 0-40 mg/L  (~ 80% viral infections) | 776 | | | **95.0** | | 41 | | **5.0** | | 817 | | 1 | |  | | 0.5815 | |  | |  | |  | |
| >40 mg/L  (~ 80% bacterial infections) | 213 | | | **95.1** | | 11 | | **4.9** | | 224 | | 0.82 | | 0.41-1.68 | |  | |  | |  | |  | |
| Malaria test result^9^ |  | | |  | |  | |  | |  | |  | |  | |  | |  | |  | |  | |
| Negative | 7 | | | **100** | | 0 | | **0** | | 7 | |  | |  | |  | |  | |  | |  | |
| Positive | 1167 | | | **95.2** | | 59 | | **4.8** | | 1226 | | na | |  | |  | |  | |  | |  | |

Abbreviations and footnotes: aOR: adjusted Odds Ratio; CI: Confidence Interval; LRT: Likelihood Ratio Test; OR: Odds Ratio; ref: reference category.

^1^p-values obtained from logistic regression likelihood ratio test, across all cases of ‘severe’ pneumonia (WHO 2005 definitions: Children presenting to hospital with cough or difficulty breathing (observed or history of) and observed LCWI but no danger signs) using ‘country’ as a forced, indicator variable. During backwards regression modelling covariates were removed from the model if they did not significantly improve the fit of the model to the data (p>0.05). When variables associated with the outcome in univariable analyses were added back into the model they did not significantly increase the fit of the model to the data. A total of 1221 observations of 1267 were used in the final model. Due to the low number of events in each country, random effects were used to control for clustering by country rather than calculating a fixed effect for each country, so individual ORs for each site were not calculable.

^2^ Oxygen saturation of 80-91% or on oxygen at admission with no oxygen saturation measurement on room air.

^3^ Hypoxaemia was defined as <90% across all sites, or oxygen requirement on admission.

^4^Hypoxiaemia was defined as oxygen saturation <90% in Zambia and South Africa (sites at altitude) and <92% at all other sites, or oxygen requirement on admission.

^5^ Raised respiratory rate at admission defined as: < 2 months of age: ≥60 breaths/min, 2-11 months: ≥50 breaths/min, 12-59 months: ≥40 breaths/min. Elevated heart rate on admission was defined as: if 1-11 months of age: >160 bpm, 12-35 months: >150 bpm, 36-59 months: >140 bpm.

^6^ Maximum duration of illness with fever, cough, difficulty breathing or wheeze

^7^ WHO classifications: very low is <-3SDs away from the mean, low is >=-3 SDs but <-2SDs away from the mean; Normal to high is >=-2SDs.

^8^Anemia was classified as per the WHO guidelines for the management of common childhood illnesses (2013 ed.).

^9^Malaria tests were not conducted in South Africa, Bangladesh or Thailand where prevalence is thought to be <1%; missing results for these countries were imputed as negative for the purposes of these univariate analyses.

**S9 Table. Comparison of risk factors for mortality identified in similar published analyses**

| **Agweyu et al.** | | **Gallagher et al.** | | |
| --- | --- | --- | --- | --- |
| **a. All pneumonia cases** | **b. Less-severe pneumonia cases** | **a. All pneumonia cases** | **Less-severe pneumonia cases** | **Less severe pneumonia** |
| i.e. pneumonia with any of the danger signs^[[1]](#endnote-1)^ and less-severe pneumonia (fast breathing +/- lower chest indrawing) | i.e. cases with fast breathing and/or lower chest indrawing but no danger signs | i.e. pneumonia with any of the danger signs^ii^ and less-severe pneumonia (fast breathing +/- lower chest indrawing) | i.e. fast breathing and/or lower chest indrawing | i.e. fast breathing and/or lower chest indrawing in 2-11-month olds only |
| **Age of child**  2-11 months vs.  12-59 months | **Age of child**  2-11 months vs.  12-59 months | **Age of child**  1-11 months vs.  12-59 months | **Age of child**  2-11 months vs.  12-59 months |  |
| **Sex**  Female vs male | **Sex**  Female vs male | **Sex**  Female vs male |  |  |
| **Malaria prevalence**  High vs low | **Malaria prevalence**  High vs low |  |  |  |
| **Respiratory rate**  ≥70 breaths per min  <70 breaths per min | **Respiratory rate**  ≥70 breaths per min vs <70 breaths per min |  |  |  |
| **Lower chest indrawing**  Present vs absent | **Lower chest indrawing**  Present vs absent |  |  |  |
| **Axillary temperature**  ≥39^0^C vs <39^0^C | **Axillary temperature**  39^0^C vs <39^0^C |  |  |  |
| **Weight for age**  <-2 to -3 SD  <-3 SD  vs ≥-2SD | **Weight for age-z score**  <-2 to -3 SD  <-3 SD  vs ≥-2SD | **Weight for *height* score**  <-3 SD  <-2 to -3 SD  vs ≥-2SD | **MUAC/ Weight for age z score**  <-3 SD  <-2 to -3 SD  vs ≥-2SD | **MUAC/ Weight for age z score** <-3 SD  <-2 to -3 SD  vs ≥-2SD |
| **Pallor**  Mild to moderate  Severe  vs absent | **Pallor**  Mild to moderate  Severe  vs absent | **NA** | **NA** | **NA** |
| **Dehydration**  Some  Severe  vs absent | **Dehydration**  Some  vs absent |  | *[Measured as skin turgor/ Capillary refill time/ diarrhoea- not significant]* | **History of diarrhoea**  Present vs absent |
|  |  | **Unresponsiveness**  Alert vs. any unresponsiveness |  |  |
|  |  | **Deep Breathing**  Present vs absent |  |  |
|  |  | **Cough (observed)**  Present vs absent | **History of cough**  Present vs absent |  |
|  |  | **Grunting**  Present vs absent |  |  |
|  |  | **Hypoxaemia**  >=92% vs. <92% | **Hypoxaemia**  <80%, 80-92% vs. >=92% | **Hypoxaemia**  <80%, 80-92% vs. >=92% |
|  |  | **Duration of illness prior to presentation**  0-2, 3-5 vs. > 5days |  |  |
|  |  |  | **HIV Exposure**  Exposed vs, unexposed |  |

1. [↑](#endnote-ref-1)
